# Indoor residual spraying for malaria control in sub-Saharan Africa 1997 to 2017: an adjusted retrospective analysis

**DOI:** 10.1186/s12936-020-03216-6

**Published:** 2020-04-10

**Authors:** Julie-Anne A. Tangena, Chantal M. J. Hendriks, Maria Devine, Meghan Tammaro, Anna E. Trett, Ignatius Williams, Adilson José DePina, Achamylesh Sisay, Ramandimbiarijaona Herizo, Hmooda Toto Kafy, Elizabeth Chizema, Allan Were, Jennifer Rozier, Michael Coleman, Catherine L. Moyes

**Affiliations:** 1 Vector Biology Department, Liverpool School of Tropical Medicine, Liverpool, L3 5QA UK; 2 Big Data Institute, Li Ka Shing Centre for Health Information and Discovery, University of Oxford, Oxford, OX3 7LF UK; 3 President’s Malaria Initiative Africa Indoor Residual Spraying Project, Abt Associates, 6130 Executive Blvd, Rockville, MD 20852 USA; 4 Monitoring, & Evaluation Department, AngloGold Ashanti Malaria Limited, AO0540595 Obuasi Mine Road, P. O. Box 10, Obuasi, Ghana; 5 Malaria Pre-Elimination Program, CCS-SIDA/MSSS, Avenida Cidade Lisboa, “Prédio Bô Casa” 1º Andar, CP 855 Praia, Cabo Verde; 6 Ecole Doctorale Des Sciences de La Vie, de la Santé et de l´Environnement (ED‑SEV), Université Cheikh Anta Diop (UCAD) de Dakar, BP 1386, Dakar, Sénégal; 7 Federal Ministry of Health (FMoH), Addis Ababa, Ethiopia; 8 Programme national de lutte contre le paludisme, Androhibe en face ENAM, BP 101, Antananarivo, Madagascar; 9 Integrated Vector Management Department, Federal Ministry of Health, Khartoum, Sudan; 10 National Malaria Elimination Centre, Chainama Hills Hospital Grounds, Lusaka, Zambia

**Keywords:** Indoor residual spraying, Malaria control, Carbamates, Organochlorines, Organophosphates, Pyrethroids

## Abstract

**Background:**

Indoor residual spraying (IRS) is a key tool for controlling and eliminating malaria by targeting vectors. To support the development of effective intervention strategies it is important to understand the impact of vector control tools on malaria incidence and on the spread of insecticide resistance. In 2006, the World Health Organization (WHO) stated that countries should report on coverage and impact of IRS, yet IRS coverage data are still sparse and unspecific. Here, the subnational coverage of IRS across sub-Saharan Africa for the four main insecticide classes from 1997 to 2017 were estimated.

**Methods:**

Data on IRS deployment were collated from a variety of sources, including the President’s Malaria Initiative spray reports and National Malaria Control Programme reports, for all 46 malaria-endemic countries in sub-Saharan Africa from 1997 to 2017. The data were mapped to the applicable administrative divisions and the proportion of households sprayed for each of the four main insecticide classes; carbamates, organochlorines, organophosphates and pyrethroids was calculated.

**Results:**

The number of countries implementing IRS increased considerably over time, although the focal nature of deployment means the number of people protected remains low. From 1997 to 2010, DDT and pyrethroids were commonly used, then partly replaced by carbamates from 2011 and by organophosphates from 2013. IRS deployment since the publication of resistance management guidelines has typically avoided overlap between pyrethroid IRS and ITN use. However, annual rotations of insecticide classes with differing modes of action are not routinely used.

**Conclusion:**

This study highlights the gaps between policy and practice, emphasizing the continuing potential of IRS to drive resistance. The data presented here can improve studies on the impact of IRS on malaria incidence and help to guide future malaria control efforts.

## Background

Malaria prevalence has declined through decades of control and treatment, with the implementation of insecticide-based vector control proving crucial [1]. Indoor residual spraying (IRS) is a key insecticide-based vector control tool for controlling and eliminating malaria in a variety of malaria epidemiological settings, yet very little information is available on which insecticides have been sprayed, where, and how much across sub-Saharan Africa. The utility of IRS as an intervention was first demonstrated during the Global Malaria Eradication Campaign (GMEP, 1955–1969) when DDT spraying in combination with case treatment, environmental management and housing improvements, decreased the global population at risk by 700 million [4, 5]. By 1978, the GMEP had resulted in the elimination of malaria from 37 countries. This success led to the expansion of IRS use in Africa where, subsequently, many IRS campaigns have been successful in controlling malaria in a range of different environments [2, 3, 6, 7].

Little is known about the large-scale deployment of IRS over the last 20 years and the impact it has had on the development of insecticide resistance in malaria vectors. In 2006, the World Health Organization (WHO) stated that countries should report on coverage and impact of IRS [4]. Regrettably, this did not result in comprehensive reporting or mapping. The available data for IRS are still limited and unspecific. Despite the important implications associated with the choice of compounds sprayed [8], studies that have considered the impact of heterogeneous IRS coverage on malaria transmission in Africa typically consider it a single intervention without distinguishing between the different insecticides used [1, 2, 9]. In contrast to insecticide-treated nets (ITNs), which primarily uses pyrethroids, the WHO has approved 16 insecticide formulations from five insecticide classes for IRS [10]. The main classes are carbamates, organochlorines, organophosphates and pyrethroids, with neonicotinoids recently added. These insecticide classes have different residual activity, cost and efficacy in the field (Table 1). Cost of the insecticide is about 30% of the total IRS campaign expenses [11]. Due to the low cost and longer residual decay rates compared to other insecticides, DDT and pyrethroids have been most popular. However, development of resistance has forced the use of alternative insecticides, which can be up to 19 times more expensive [12]. Although the prices have dropped in recent years [13], these new compounds are still more expensive than DDT and pyrethroids. The various insecticide classes have different residual activity, impact on local malaria vector populations, cost implications and levels of social acceptance [8]. This has important implications for local malaria vector populations and consequently local malaria epidemiology [3, 4]. The insecticide choice can also drive resistance to the compound used, and to other insecticide classes via cross-resistance [14]. The different IRS formulations can thus be considered as distinct control methods.

**Table 1:** The main classes of insecticides used for IRS

Insecticide class	Representative compounds	Residual activity (months) [10]	Proportional costs^a^	Insecticide resistance in sub-Saharan Africa [49, 50]^b^
Organochlorines	DDT	6 to 12	1	+++
Pyrethroids	Alpha-cypermethrin, deltamethrin, lambda-cyhalothrin	3 to 6	2.5	+++
Organophosphates	Malathion, pirimiphos-methyl	2 to 6	6	+
Carbamates	Bendiocarb, propoxur	2 to 6	10	++
Neonicotinoids	Clothianidin	3 to 8	ND	–

*ND* no data

^a^Proportional costs to spray 250 m^2^ at WHO recommended target dosage according to data obtained from Oxborough, 2016 [16]. Costs are exemplary and exclude costs of shipping, disposal of insecticides and environmental precautions

^b^Malaria vector resistance spread in Sub-Saharan Africa, identified by standard WHO susceptibility tests (-) unknown, (+) < 25% tests show resistance, (++) 25–50% tests show resistance, (+++) > 50% tests show resistance

The emergence and spread of resistance to organochlorines and pyrethroids has led to policy recommendations to switch to alternative insecticides for IRS [3, 15] often at an increased cost [16]. In 2012, the WHO’s Global plan for insecticide resistance management in malaria vectors (GPIRM) recommended: i) pre-emptive use of annual rotations of insecticide classes with differing modes of action if resources allow for it, ii) avoidance of pyrethroid IRS in areas of high long-lasting insecticide-treated net (LLIN) coverage, and iii) focal IRS with a non-pyrethroid insecticide in pyrethroid resistance hotspots [15]. Unfortunately, the uptake of GPIRM is unclear in many areas [17]. There is a need for detailed data on IRS use to understand the full extent of the gap between policy and practice. This is especially pressing since insecticide resistance has the potential to derail malaria control [18]. It is important to remember the GMEP success, but not forget that un-sustained control efforts can result in a resurgence of malaria incidence [16, 19, 20]. The aims of this study were to estimate the spatiotemporal coverage of IRS for sub-Saharan Africa for each of the four main insecticide classes (carbamates, organochlorines, organophosphates and pyrethroids) that were used from 1997 until 2017, to identify trends in IRS activities throughout Africa and to assess the response to the Global plan for insecticide resistance management in malaria vectors.

## Methods

### Overview

IRS coverage for the 46 malaria-endemic sub-Saharan African countries was calculated for the years from 1997 to 2017 at the most spatially disaggregated level possible, which ranged from local authority (sub-district) to country level data. The data obtained encompassed a range of measurement types that were adjusted to one standard unit of measurement: the proportion of households sprayed.

### Data sources

Data on IRS campaigns were collected using reports from National Malaria Control Programmes, Ministries of Health, the President’s Malaria Initiative (PMI), WHO, and other stakeholders (Table 2). Data on IRS campaigns managed by PMI from 2012 to 2017 were shared by Abt Associates. Published reports on IRS were identified using the Google search engine and the search terms “indoor residual spraying”, “IRS” and, depending on the main language in the country, “pulvérisation intradomiciliaire” (French) or “Pulverização Intradomiciliária” (Portuguese) for each country. If data were incomplete, additional information was requested from stakeholders. Published articles from 1997 to 2018 were identified in the Web of Science bibliographic database using the search terms “indoor residual spraying” with the name of each sub-Saharan African country in turn. Only studies that described an IRS campaign were retained, giving 366 articles out of 1,036. This study aimed to provide a continent-wide overview of IRS activities and therefore, data that did not describe a mass spray campaign encompassing multiple settlements, such as hut trials, were excluded.

**Table 2:** Summary of data sources used to calculate IRS coverage

	Subnational					National					Confirmed no spraying
	Households sprayed	Structures sprayed	People protected	Insecticide quantity	Unknown	Households sprayed	Structures sprayed	People protected	Insecticide quantity	Unknown	
Spray reports		102			1		17	1		3	110
Stakeholders report		3	5				3	1		3	7
National malaria control programmes	4	19	1								19
Personal communication		47	6							22	155
Literature	1	28	5	5	18	1		3		25	99
DHS	13										2
WHO reports		7	11				19	42	3	10	89
MAP data					11					30	

Authors of reports and scientific publications who were based at or worked with the National Malaria Control Programmes (NMCPs) were contacted. Data were requested on which areas were sprayed, how many households were sprayed, what insecticide compounds were used, and confirmation was obtained on the times and places when there was no IRS. It was assumed that the NMCP in-country signed off on all IRS activities and would, therefore, be aware of all spraying activity. Data on whether occupants reported that their house had been sprayed in the previous 12 months was downloaded from the Demographic Health Surveillance (DHS) Program website for 128 country-years [21]. The proportion of households that answered ‘yes’ to the question ‘Has your house been sprayed in the last 12 months’ was used as the proportion of households sprayed. Finally, data on the number of people protected (residents of households that were sprayed) were obtained from the WHO World Malaria Reports. Data sourcing and follow up was completed on 1 December 2018. In total, 65 scientific articles, 57 reports and 16 unpublished data sets were collated. Any duplicates were removed. The aim was to collect a total of 21 years of data for 45 different countries and 7 years of data for South Sudan (2011–2017), totalling 952 country-years.

Data is presented for 951 country-years. If data was collated from multiple sources, the main source is represented. Household is a person or group of people that live and eat together; Structure is a permanent building with a roof and walls, such as houses, sheds and animal shelters; People protected is defined as the residents living in households that were sprayed; Insecticide quantity is the amount of insecticide used to spray an area (in kilogram or litres). Spray reports are spray campaign reports published by the organization conducting the spray campaigns; Stakeholder reports are reports from secondary sources on spray campaigns; NMCPs are governmental organizations that oversee the malaria control activities in country; Personal communication is information received through personal contact with stakeholders, such as members of the NMCP, malaria researchers and non-governmental organization employees in-country. Literature is published scientific literature. WHO reports are reports accessed through the World Health Organization webpage. The MAP data is data collated by the Malaria Atlas Project freely accessible through their webpage.

### Linking IRS data to administrative divisions

IRS coverage data were reported for countries and first, second and third order administrative divisions (provinces, districts, subdistricts). The smallest administrative division for which IRS data were available was used. Publicly available data on administrative division boundaries (in shapefile format) were used wherever possible, principally the FAO’s Global Administrative Unit Layers (GAUL) [22] and the Database of Global Administrative Areas (GADM) [23]. Administrative divisions were matched using the division name and any available maps showing its boundaries. In instances where these boundaries did not match the area sprayed, national boundary files that did match the IRS data were sourced. Finally, a new shapefile was constructed for the Zambian districts that were used by the NMCP from 2014 onwards [24, 25]. The DHS data were provided with GPS coordinates for household clusters. Each cluster was linked to a second order administrative unit from GAUL using the QGIS Geographic Information System software, version 3.2.3.

### IRS data adjustments

The different data sources did not use one consistent measure of IRS coverage. Where available, data on the number of households sprayed, structures sprayed, people protected, and insecticide quantity were converted to the standard measurement ‘proportion of households sprayed within the administrative division (${\hat{p}}$)’, to provide IRS coverage values that could be compared across areas of different sizes. The proportion of households sprayed, and proportion of people protected were assumed equal (${\hat{p}}_{h} = {\hat{p}}_{p}$). If quantitative data on IRS use were not available from the groups managing IRS campaigns, indirect data sources (DHS and WHO World Malaria Reports) were used.

#### Using the number of households sprayed

The proportion of household sprayed in an administrative unit (${\hat{p}}_{h}$) was calculated using formula (1).

1 $${\hat{p}}_{h} \, = \,\frac{{X_{h} }}{{n_{h} }}$$

${\hat{p}}_{h}$ = proportion of households sprayed in an administrative division $X_{h}$ = number of households sprayed in an administrative division $n_{h}$ = number of households in an administrative division.

The number of households in an administrative unit was calculated by dividing the population of the administrative unit by the average number of people per household for that area. The population data were obtained from WorldPop [26]. Subnational values for the number of people per household were obtained from the GDL dataset [27], and for instances where subnational values were not available, national values were obtained from the United Nations Database of Household Size and Composition [28].

#### Using the number of structures sprayed

The proportion of households sprayed in an administrative unit (${\hat{p}}_{h}$) was calculated using formula (2).

2 $$\hat{p}_{h} = \left( {\frac{{X_{s} }}{{n_{h} }}} \right)\left( {\frac{1}{{\hat{p}_{{\max }} }}} \right),\quad if\,\, \hat{p}_{{\max }} > 1$$

$\hat{p}_{h}$ = proportion of households sprayed in an administrative division $X_{s}$ = number of structures sprayed in an administrative division $n_{h}$ = number of households in an administrative division $\hat{p}_{max}$ = the maximum value obtained for $X_{s}$/$n_{h }$ in any administrative division in that country.

The number of structures in an area does not necessarily equal the number of households in that area, however, “structures” were often not defined by the data sources and no data are available for the number of structures per household within an area. For instances when the number of structures sprayed was greater than the number of households present, the highest ratio of structures to households in any administrative divisions was used to adjust the proportion of households sprayed, calculated for all administrative divisions from the same data source and country. To limit underestimation, each adjustment was calculated using data from a single country and source, with the assumption that data from a single source were compiled using consistent methods and the adjustment could, therefore, be applied to all data supplied by that source for that country. For instances where the number of structures sprayed never exceeded the number of households in any part of the country, no adjustment was made.

#### Using the number of people protected

If neither the number of households sprayed, nor the number of structures sprayed was available, the proportion of population protected was calculated using formula (3). It was assumed that $X_{p}$ is equal to $n_{p}$, because spray campaigns do not spray entire administrative division by default but typically target specific areas or house structures within the administrative division. People protected is defined as the residents living in households that were sprayed. The estimate of $\hat{p}_{p}$ is equal to the estimate of $\hat{p}_{h}$, as the calculation cancels out the ‘number of people per household’.

3 $$\hat{p}_{p} =\,\frac{{X_{p} }}{{n_{p} }}$$

$\hat{p}_{p}$ = proportion of population protected in an administrative unit $X_{p}$ = number of people protected in an administrative unit $n_{p}$ = number of people in an administrative unit.

#### Using insecticide quantities

If only the quantity of insecticide used during a spray campaign was available, the data were converted into the estimated number of structures sprayed ($X_{s}$). The conversion rate was calculated using spray campaign data, which presented both the number of structures sprayed and the amount of insecticide used. The average number of structures sprayed per kg/l insecticide (the conversion rate) was calculated separately for every country and insecticide formulation. If the data were unavailable within a country, the average conversion rate (4.74 structures per kg DDT) for an insecticide formulation throughout the database was used. The proportion of households sprayed ($\hat{p}_{h}$) was then calculated using the estimated number of structures sprayed (formula 2).

#### Calculating IRS coverage using indirect sources

If campaign data were not available, DHS data were used. Second order administrative divisions statistics were derived from cluster-level data for 128 country-years. In a small number of surveys, the survey did not cover all second order administrative divisions within a country. In those instances, the national proportion of households sprayed was extracted and used to fill the gaps. In some instances, data for a country-year were available from both direct sources and DHS, and the two sets of values were compared to identify any adjustment required for instances when only demographic survey data were available. No consistent trends were seen (for example, in 2012 the DHS values were 3.2 times higher in Senegal and 1.4 times lower in Burundi than the values from direct sources) so no adjustment was applied. Any remaining gaps in IRS coverage were filled using national-level coverage data from the WHO World Malaria Reports and the Malaria Atlas Project’s database [1, 29].

### Identification of insecticide classes sprayed

The insecticide formulations used were recorded and classified as carbamates, organochlorines, organophosphates or pyrethroids. If respraying occurred within 6 months using the same insecticide class, only the highest coverage value was included. If several insecticides were used in one country in 1 year and separate quantities were not provided, it was assumed that they were used in mosaic without overlap and the total IRS coverage value was equally divided between the insecticide formulations used. The indicator for possible rotation was based on the national use of insecticides, with the assumption that policy on insecticide-use is made nationally. Data on possible rotation of insecticides was derived by comparing the insecticide classes used by each country in each year from 1997 to 2017. Data was excluded if in either year no spraying occurred, or insecticide class was unknown. No rotation was assumed if (1) one or more insecticide classes were the same as previous year with no addition of a new class and (2) pyrethroids were substituted for organochlorines or the other way around (cross-resistance). There was indication of rotation if (1) insecticide classes were different from previous year or (2) if a new insecticide class was added to the existing class(es) [15].

### Construction of map data layers

Once values for the proportion of households sprayed had been generated and linked to the appropriate administrative division, a shapefile for each insecticide class and year was constructed using the geographic information system ArcGIS. Each shapefile consisting of a mosaic of third, second, first and zero order administrative divisions was then rasterized to provide IRS coverage values for every cell in a 2.5 arc-minute (~ 5 x ~ 5 km) grid cell in GeoTIFF format.

In instances where it was known that IRS was deployed but no quantitative data were available to calculate the coverage achieved, the gaps in the mapped data were filled using data from the same admin unit 2–3 years before and/or after. If these data were not available, the average IRS coverage for that year was used to fill in the gaps.

### Calculating number of people protected

Maps of the ‘proportion of households sprayed’ show spatial variation in IRS coverage using a metric that is consistent across space for units with differing areas, but the metric of ‘proportions of households sprayed’ needs to be converted to the ‘number of people protected’ in order to see trends through time in the absolute number of people protected. Once a complete set of maps, with no data gaps, were available, the proportion of households sprayed was transformed into the number of people protected using WorldPop’s population rasters for 2000 to 2020 [30]. Population rasters for the earlier years, 1997 to 1999, were calculated using the population raster from 2000 and national UN growth rates for the period 1995-2000. The number of people protected by IRS was calculated by multiplying the IRS coverage rasters by the population rasters for each year, that is the proportion of households sprayed was multiplied by the number of people. Similarly, the ITN values were calculated by multiplying the ITN rasters [30] by the human population rasters.

## Results

### Geospatial data on IRS coverage across Africa, 1997–2017

A total of 21 years of data for 45 different countries and 7 years of data for South Sudan (2011–2017) were collected, totalling 952 country-years. Absence of IRS campaigns were confirmed in 50.3% of country years. Congo in 2017 was the only country-year for which presence or absence could not be confirmed. Coverage values for the remaining 472 country years were collected at various aggregation levels giving 6029 administrative division years. For seven country years, the insecticide class used for spraying could not be identified (coloured grey in maps), while for 82 country years spraying with a specific insecticide class were identified but IRS coverage could not be quantified (hatched in maps). It is important to note that some sprayed areas are too small to be evident on the map images, however, the raster files can be zoomed to an area of interest in any GIS software (available through the Figshare repository [31, 32]).

### Trends in IRS use from 1997 to 2017

**Fig. 1:**
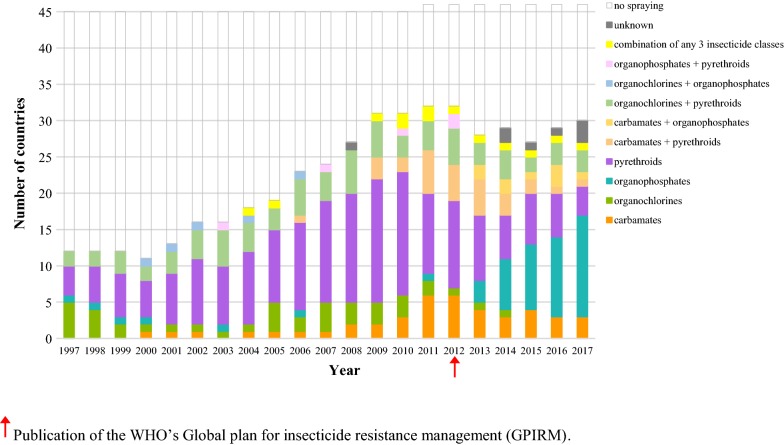
The insecticide class or combination of classes used for indoor residual spraying in the 46 malaria-endemic sub-Saharan countries over time

Figure 1 shows how the number of countries deploying IRS has increased considerably over the years and the changes in mapped coverage through time are shown as an animation (available through Figshare [33]). About 26% of sub-Saharan African countries deployed IRS in 1997 (12/45), which by the end of 2017 had increased to 63% (29/46). Within countries, coverage ranged from small pilot studies to mass country campaigns. From 1997 to 2005 there was a steady increase in IRS coverage and spraying mainly occurred in the south and north-east of Africa. There is a clear increase in IRS activity from 2005, the year that PMI launched their IRS programs. The highest IRS coverage was achieved in 2010–2013, partly due to the initiation of IRS in West Africa.

### Subnational variation in IRS coverage

**Fig. 2:**
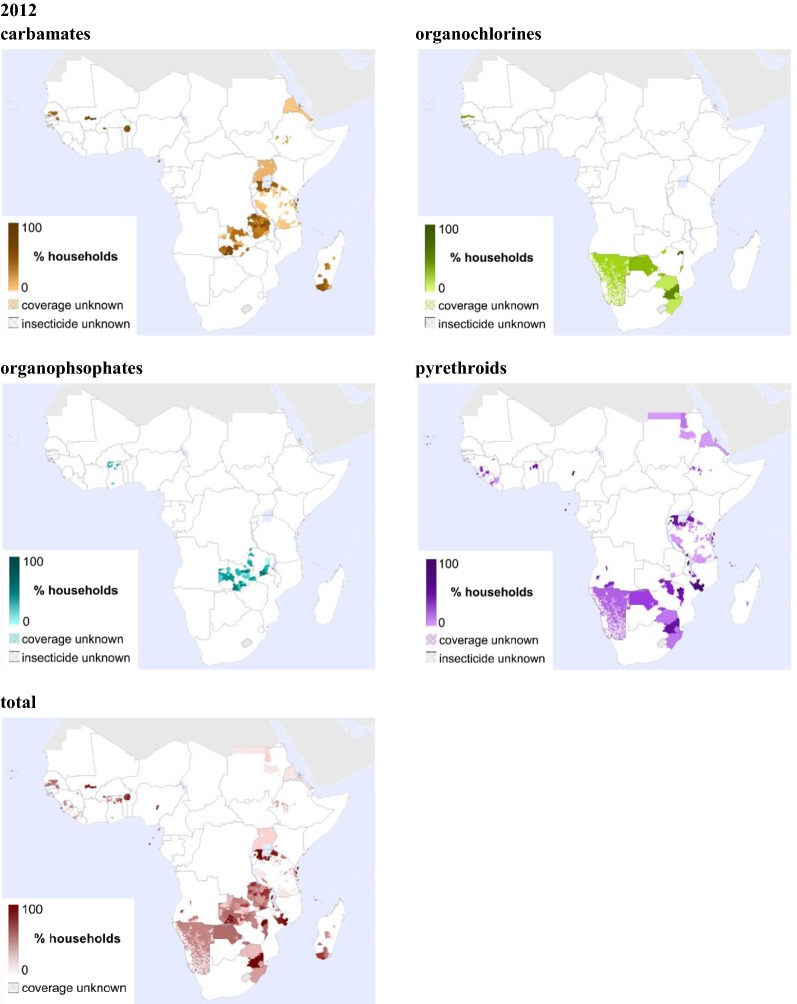
Maps showing IRS coverage in sub-Saharan Africa **i**n 2012

Subnational variation (where subnational data were available) is visualized for the full study period in Additional file 1 and the animation. One example year is highlighted, year 2012, when IRS coverage was highest (Fig. 2). Insecticides from all four classes were used in sub-Saharan Africa in 2012. Interestingly, the high number of countries implementing IRS did not translate into a high percentage of land area covered, with Zambia and Rwanda clear exceptions. The difference in data aggregation, from third-order to national-level, heavily influences the coverage values presented. Where high proportional cover may have been achieved within a target area but, without any information on the boundaries of that target area, the proportional cover for the wider administrative division was calculated. If no information was available for which administrative divisions encompassed target areas, the proportional cover across the whole country was calculated resulting in lower proportional coverage values across a wider area. These biases are present in all years and highlight the necessity to keep improving IRS databases for future use. If the IRS coverage data disaggregated to third-order level only are considered, 3.2% of areas reached 80% coverage (13/399 subdistrict years). Low coverage was sometimes associated with the use of two or more formulations. For example, in Tanzania in 2012 carbamate and pyrethroid use overlapped, with less than 80% coverage for the insecticides individually, but higher total coverage. In another example, in Mjini region 0.48 proportion of houses were sprayed with carbamates and an additional 0.48 proportion of houses with pyrethroids.

### National-level choices of insecticide for IRS

Figure 1 shows that all countries relied heavily on organochlorines (DDT) and pyrethroids (alpha-cypermethrin, deltamethrin and lambda-cyhalothrin). A slight peak in organochlorine use can be seen the year after DDT was approved for vector control by the Stockholm Convention in 2004. At this time, carbamates were only used on Bioko island in Equatorial Guinea and in small areas of Mozambique and South Africa. Similarly, organophosphates (malathion) were only sprayed in Comoros, Eritrea and Sudan.

Although the choice of organochlorine remains consistent, the selection of pyrethroids increases, with a peak number of countries using it in 2010. Many different pyrethroid insecticides and formulations are used during this period. There is a decline in DDT and pyrethroid use from 2010-2017, with an increase in carbamates and organophosphates from 2011. In 2017, 15 countries deployed organophosphates compared to 3 countries in 2011.

Throughout the decades, the use of just one insecticide class for IRS activity within a country was common. Mono-treatments were implemented in 70% (332/473) of the country-years where IRS was implemented. In 27% of the country-years (128/473), two classes were used in-country simultaneously. This was often a combination of organochlorines and pyrethroids (77/128). The use of these classes in an area were sometimes related to the different wall surface types, with pyrethroids sprayed on painted walls due to the visible residues left when DDT is sprayed [34]. In 3% of cases, three different insecticide classes were used in-country simultaneously (13/473). Most notable is South Africa, where IRS using carbamate, organophosphate and pyrethroid was deployed in 5 years.

**Fig. 3:**
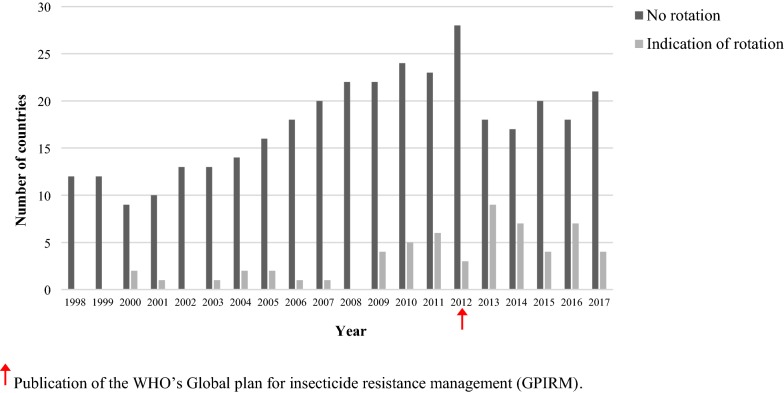
Possible rotation between the insecticide classes used for indoor residual spraying by country from 1998 to 2017

Data on possible rotation activity for the different countries from 1998 to 2017 were derived (Fig. 3). Since 2000, there is evidence for the use of rotations in those countries that used IRS in consecutive years but a far greater number did not rotate insecticide classes throughout the study period. There is an increase in the evidence for rotation activity in 2013, the year following the publication of the GPIRM, although this does not persist. However, there is also a noticeable decrease in the number of countries using the same insecticide class for IRS in consecutive years from 21-28 countries per year in 2008–2012 to 17–21 countries per year in 2013–2017.

### Number of people protected by IRS with each insecticide class

**Fig. 4:**
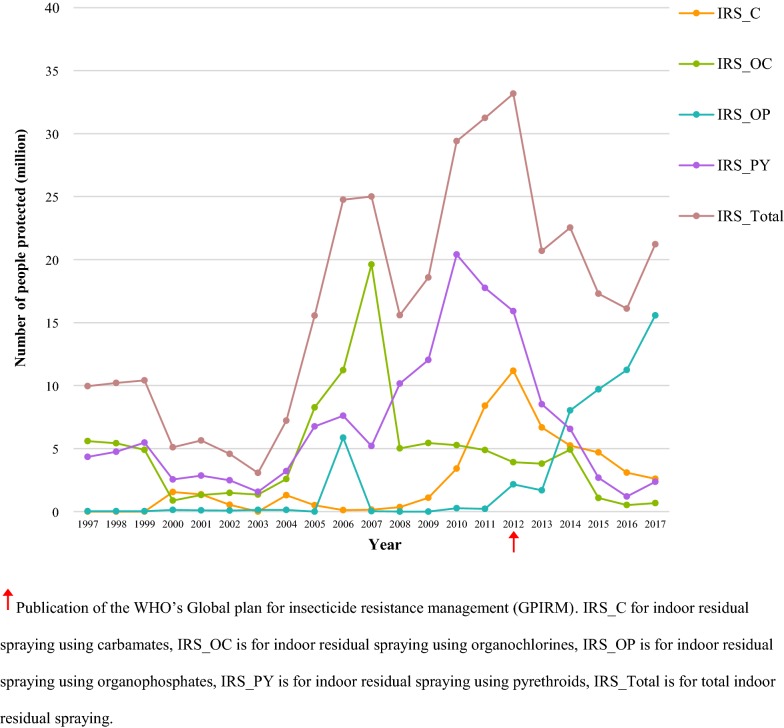
Estimated number of people protected by carbamate, organochlorine, pyrethroid, and organophosphate IRS from 1997 to 2017

The number of people protected by IRS for the different insecticide classes shows a similar trend in choice of insecticide as previously discussed at the national level (Fig. 4). Namely, organochlorine use increased after 2004, peaked in 2007 and has since dropped to very low levels but persists. Pyrethroids and organochlorines protected most people until 2014, two years after the publication of GPRIM, when a concurrent increase in people protected by organophosphate IRS can be observed. The total number of people protected by IRS increased in each year from 2004 to 2006 and has maintained similar coverage levels since then.

**Fig. 5:**
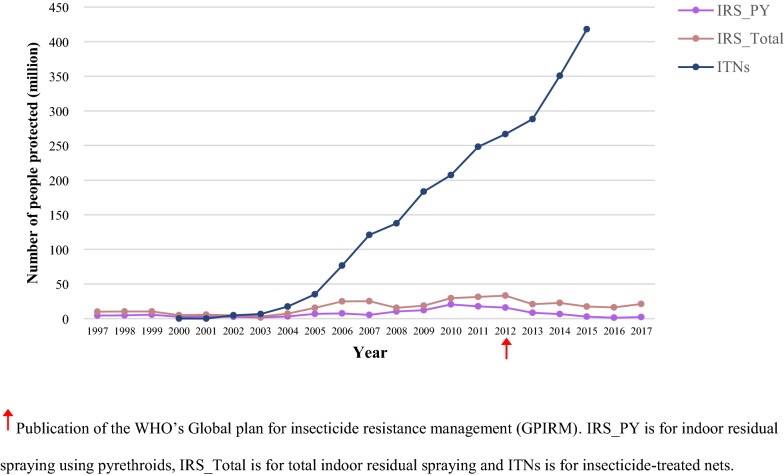
Estimated number of people protected by pyrethroids in ITNs and IRS from 1997 to 2017

Since the mass introduction of ITNs from 2002 onwards, nets have been the most prevalent method for protecting people from malaria vectors (Fig. 5). At no point did IRS coverage reach as many people as ITNs did. The trends shown in the number of people protected indicate that use of pyrethroids in IRS is far outweighed by the deployment of pyrethroid-treated nets.

To limit resistance development, GPIRM recommends avoiding the use of pyrethroid IRS in an area with high ITN cover [15]. With IRS activity now differentiated between the insecticide classes, it is possible to identify areas where overlap in pyrethroid-based vector control has occurred. Comparing the modelled ITN coverage [35] (Additional file 2) and pyrethroid IRS coverage (Additional file 1) shows that overlap in pyrethroid-based vector control has occurred. In 2000, no ITNs were distributed and there was limited pyrethroid IRS. Five years later ITNs were used throughout the centre of sub-Saharan Africa, some areas in west Africa and the parts of Madagascar. Conversely, pyrethroid IRS was implemented in other areas of Madagascar, Botswana and South Africa, where ITNs were not distributed. Only minor overlap was seen in Angola, Kenya, Mozambique, Rwanda and Zambia. In 2007, almost all countries in sub-Saharan Africa had some ITN coverage except Nigeria, Mauritania, parts of DRC and southern Africa (Namibia, Botswana and South Africa). Again, pyrethroid IRS was most intense in southern Africa, where ITNs were not distributed. There was overlap in pyrethroid use in neighbouring countries (Zambia and Angola), with other small areas of overlap throughout Africa. In 2010, all regions except southern Africa were, in part, protected by ITNs, with overlap with pyrethroid IRS apparent in at least 17 countries. In 2015, the extent of ITN coverage was similar to 2010, albeit more intense, with pyrethroid IRS use declining. Pyrethroid IRS was mainly implemented in the southern countries where ITNs were not distributed. Only minor overlaps were seen in Somalia, Eritrea, Sudan, Malawi and Mozambique.

### Data availability

The datafile in Excel format (Microsoft Office 365 ProPlus) and IRS coverage rasters are shared through the Figshare repository [31, 32]. The datafile includes the year, country, most disaggregated administrative level, shapefile source with the unique identifier for each admin unit, IRS activity, insecticide brand name, insecticide formulation, insecticide class, proportion of structures sprayed, raw data type, source for coverage data, source for insecticide class data and a note of whether the raw data are publicly available. If the data for insecticide brand name, insecticide formulation, insecticide class or proportion of structures sprayed were unknown, they are marked as such. Rasters at a 2.5 arc-minute (approximately 5 km) resolution are provided for each insecticide class and for total IRS, for each year. Each raster provides values for coverage from 0 to 100% and unknown values are represented as no-data.

## Discussion

This paper is the first to present the spatiotemporal distribution of IRS coverage separately for each of the four main insecticide classes throughout sub-Saharan Africa from 1997 to 2017. In the past, coverage values have been presented at the national level only and IRS has been described as a single intervention method, whilst the different insecticides can have profoundly different impact on local malaria vector population and thus malaria transmission dynamics [8]. This paper quantifies IRS activities for each insecticide class to examine spatial and temporal trends in IRS deployment and elucidates whether insecticide resistance management guidelines have been followed.

The results highlight a doubling of the number of countries using IRS since 1997. The high number of countries implementing IRS did not translate into high land area covered or high numbers of people protected, reflecting the highly focal nature of many IRS campaigns. It is important to note that the coverage values differ from PMI coverage values because this paper uses ‘all households in an administrative division’ as the denominator to define the proportion of households sprayed, while PMI uses ‘targeted households’ as the denominator and the number of ‘targeted households’ was often not given. Compared to ITNs, only a small number of people have been protected from malaria vectors by IRS. The choice of where to spray in-country is, in part, dependent on malaria incidence, although insecticide resistance, financial resources, accessibility and politics may play a more important role. Remarkably, when maps are compared with *Plasmodium falciparum* infection prevalence [1] (Additional file 3), the majority of the high malaria burden areas in west African countries and the Democratic Republic of Congo were not targeted with IRS before 2010. IRS was only deployed over small areas because significant investment, good management and well-trained staff are required [3]. This is generally more challenging than mass ITN distribution schemes across large areas of a country, and typically requires a huge amount of sustained investment. Additionally, the WHO currently recommends focal IRS in low and moderate endemic areas rather than of blanket spraying [36], although this recommendation might change in the near future [37].

IRS coverage has never been presented at this level of spatial detail and this study covers the whole of Africa for the last 21 years. A limitation while creating the database and maps was that IRS data were not collected consistently through space and time. The maps are not always representative of subnational variation in coverage, especially pre-2000. No data were available on the total number of structures in each area, so number of structures was assumed equal to the number of households unless the data indicated otherwise, meaning coverage could have been over-estimated in some areas. The DHS data used to estimate IRS coverage is retrospective data collected through surveys, which has recollection biases. Data on insecticide sales was not available to cross-check the database generated by this study. The aim of this study was to capture large-scale IRS campaigns encompassing multiple settlements, often managed by the NMCP or international NGOs. IRS targeted to single buildings, such as spraying in hospitals, was not captured. Nor was the difference in quality of IRS campaigns. Moreover, fine-scale variations due to differences in compounds, formulations, local environment, housing types, spray substrate, spray practices (e.g. mosaic spraying) and quality give rise to different residual activities on wall surfaces [38]. This was also not captured. For example, in Botswana a distinction was made between modern houses (made from cement) that were sprayed with pyrethroids, and traditional houses (made from mud) that were sprayed with DDT [39]. Although IRS is important as a community-level intervention, community protection values were not calculated (i.e. the assumption that ~ 80% IRS coverage in an area protects the remaining 20%), to limit the risk of overestimation. Furthermore, this study focussed on malaria vector control only, and IRS conducted to control other vector-borne diseases was not included.

The WHO’s GPIRM recommends that IRS with pyrethroids should not be deployed in locations where ITNs are used for malaria control, and that IRS should always rotate between insecticide classes with different modes of action. The results presented here show that countries using IRS have relied heavily on organochlorines and pyrethroids. Scaling up of IRS from 2005 until 2010 was only feasible with the availability of these insecticides. These insecticide classes are popular as they are inexpensive with long residual action [10, 40]. Insecticide resistance is one of the biggest challenges for current vector control programs and there is strong evidence that locations with high DDT resistant are also highly pyrethroid resistant [14, 18]. Resistance and cross resistance to organochlorines and pyrethroids in malaria vectors were documented well before 1997 [41], but until recently IRS campaigns were heavily dependent on these compounds. The extensive use of both classes and the dependence on pyrethroid-treated bed nets has considerable potential to drive organochlorine and pyrethroid resistance in malaria vectors.

The results show limited evidence that insecticide classes have been rotated from year-to-year, although after the publication of GPIRM a small increase in the evidence for rotation activity is visible. This was likely driven by a better understanding of pyrethroid resistance, which coincided with the publication of GPIRM, and the availability of a new long-lasting organophosphate. The lack of implementation of rotations is related to the costs and organizational challenges associated with IRS campaigns. However, the multi-country decisions by PMI to change from pyrethroids to carbamates and organophosphates, resulted in key shifts in insecticide classes used for IRS in Africa [16]. An increase in bendiocarb (carbamate) use is seen from 2011 to 2013. In 2013, a second larger shift in insecticide classes is seen with the introduction of the long lasting organophosphate pirimiphos-methyl capsule suspension formulation (Actellic 300CS. Within the space of 2 years, this formulation of organophosphates became one of the most popular insecticide classes for IRS due to its long residual activity and minimal resistance in malaria vectors. The change from the older cheaper formulations of insecticides to the newer more expensive ones has come at a financial cost [11, 16]. In 2014, the PMI calculated that for pyrethroid IRS campaigns the insecticide cost was only 6% of the total cost, while for organophosphate IRS campaigns 37% was spent on the purchase of insecticides [42]. The costs increase further for carbamates (e.g. bendiocarb formulations) when used in areas with a > 3 months transmission season, which would require two spray rounds in one transmission season. It is important to note that new insecticide formulations are generally more expensive than old formulations due to the initial development cost. The new products are likely to become more affordable over time. The cost increase in insecticides resulted in reduced IRS coverage and withdrawal in parts of Benin, Mali, Tanzania and Uganda that may have resulted in a malaria resurgence [16, 43].

The exponential growth in people protected by ITNs compared to IRS highlights the challenges associated with deploying IRS, which requires regular re-spraying, is expensive and labour intensive. The exclusive use of pyrethroids on bed nets in the past has made the implementation of pyrethroids for any other vector control method controversial. The maps show that overlap between ITNs and pyrethroid IRS has occurred until as recently as 2015, mostly in southern and north-eastern Africa. Unfortunately, disaggregated data is missing for countries such as Angola, Somalia, Sudan and Angola to identify the extent of the overlap. Reassuringly, pyrethroid and organochlorine IRS use is most common in countries where ITNs are not mass-distributed (i.e. South Africa, Namibia and Botswana).

To sustain IRS coverage, insecticide formulations need to improve in residual activity and decrease in costs. More importantly, focus should be on the development of new insecticides with different modes of action [4, 17]. This is essential as there are reports of malaria vector populations that show resistance to all four insecticide classes [44]. As of April 2019, the WHO has approved the use of 16 insecticide compounds and formulations [10, 45]. This includes the newly approved neonicotinoid clothianidin and in the future may include pyrrole chlorfenapyr [46, 47]. The availability of new products and the reduction in IRS product prices could shift current trends and increase coverage in the future.

The results presented here provide insight into spatial and temporal trends in IRS deployment and have allowed us to investigate whether GPIRM guidelines are being followed. The IRS coverage maps can also feed into future work on insecticide resistance management and malaria control. The GPIRM recommends focal IRS with a non-pyrethroid insecticide should be introduced in addition to LLINs in areas that are resistance hotspots. Once insecticide resistance maps become available, it will be possible to ascertain whether focal IRS has been deployed in resistance hotspots and to investigate what happens in hotspots where LLINs alone or LLINs in combination with IRS have been deployed [48]. The results provided here also mean that it will be possible to investigate the relative roles of ITN use and pyrethroid or organochlorine IRS use in the development of resistance. Ultimately the aim of vector control, including resistance management, is to prevent transmission of the malaria parasite. Previous studies that have investigated the heterogenous impact of ITN and IRS use over space and time have considered IRS as a single intervention type without distinguishing between the different insecticides used [1, 2, 9]. The maps provided by this study now allow malaria models to incorporate data on the different types of IRS and evaluate the role they have played.

## Conclusion

This paper presents an overview of IRS activities across Africa over the last two decades. For the first time, the four insecticide classes used have been mapped separately in time and space, and subnational variation has been captured wherever possible. Separating the insecticide classes is particularly important for studies of resistance because resistance mechanisms are typically specific to one, two or three classes [14]. This paper has shown that IRS deployment since the publication of GPIRM has often avoided overlap between pyrethroid IRS and ITN use, although this is not always the case, and that annual rotations of insecticide classes with differing modes of action are still not routinely implemented 5 years after GPIRM was published.

## Supplementary information

**Additional file 1:** Maps showing IRS coverage in sub-Saharan Africa from 19972017 for carbamates, organochlorines, organophosphates and pyrethroids.

**Additional file 2:** Maps showing modelled insecticide-treated bed net (ITN) coverage from Bhatt and Gething [35].

**Additional file 3:***Plasmodium falciparum* infection prevalence from Bhatt et al. [1].

## Data Availability

The datasets supporting the conclusions of this article are available in the Figshare repository, [https://figshare.com ID-10066643] and [https://figshare.com ID-10066910] [31, 32].
