# Supplementary material for: Indoor residual spraying for malaria control in sub-Saharan Africa 1997 to 2017: an adjusted retrospective analysis
Source: Malar J. 2020 Apr 10;19:150. doi: 10.1186/s12936-020-03216-6 (PMC7149868; doi:10.1186/s12936-020-03216-6)

Maps showing IRS coverage in sub-Saharan Africa from 1997-2017 for carbamates, organochlorines, organophosphates and pyrethroids

1997

carbamates

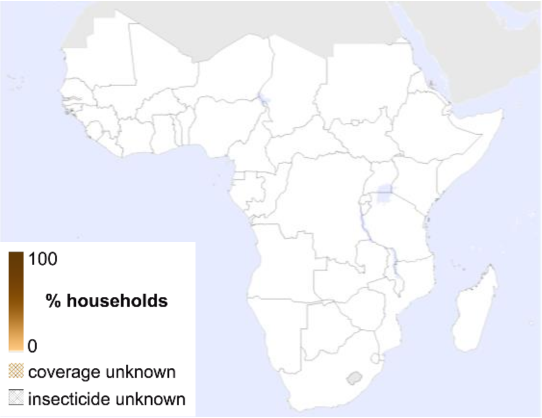

organochlorines

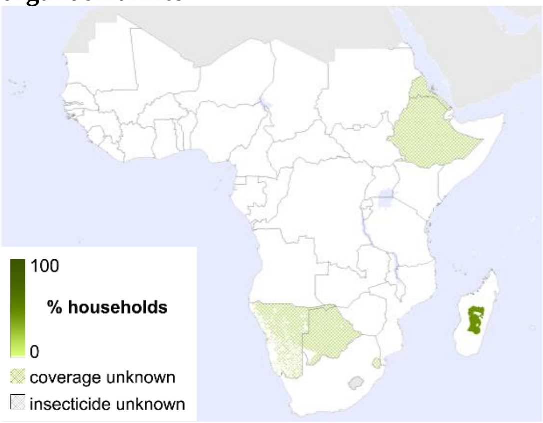

organophosphates

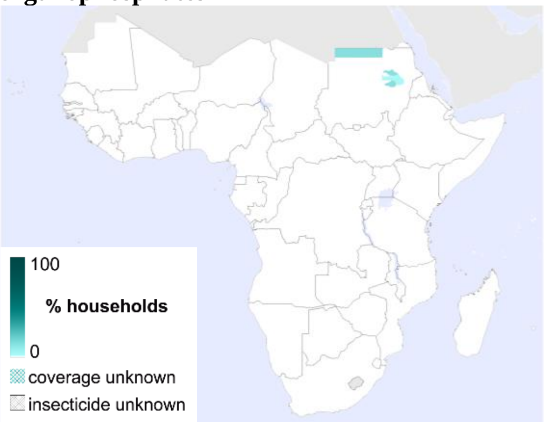

pyrethroids

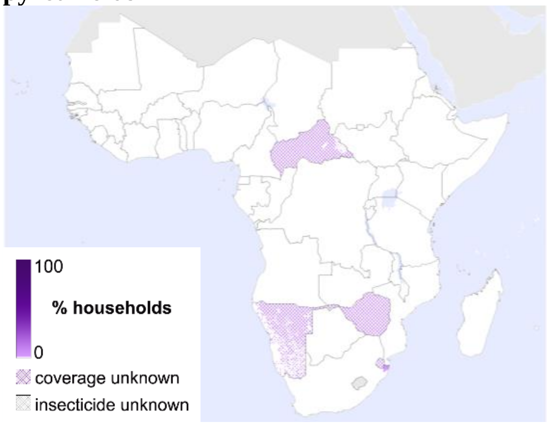

total

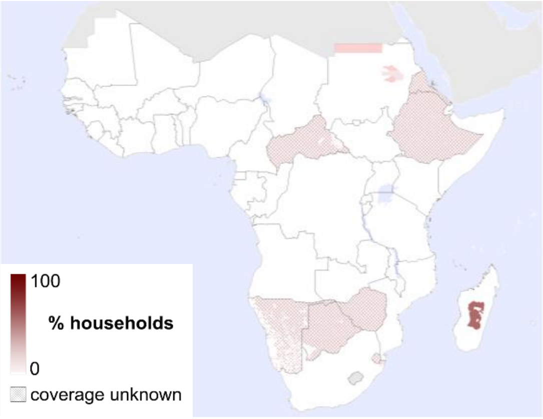

1998

carbamates

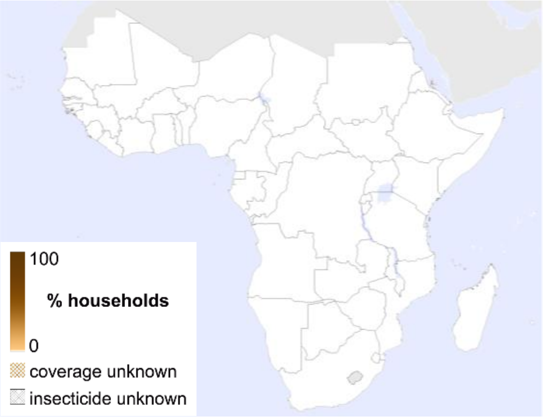

organochlorines

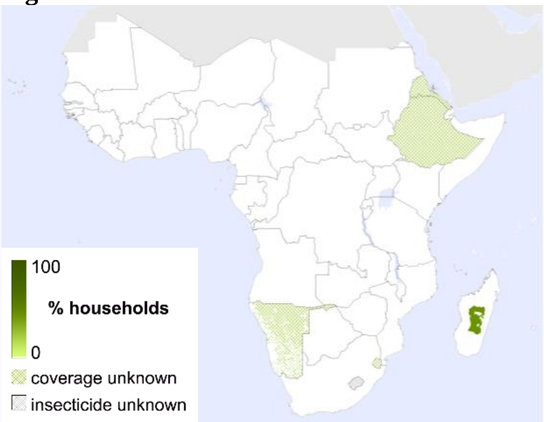

organophosphates

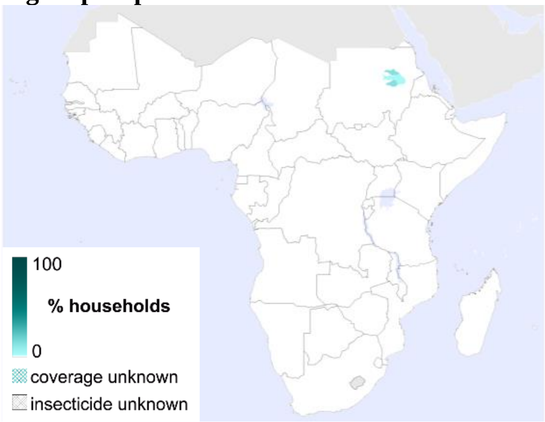

pyrethroids

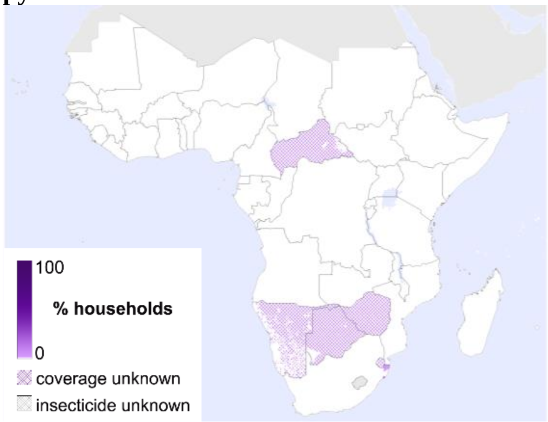

total

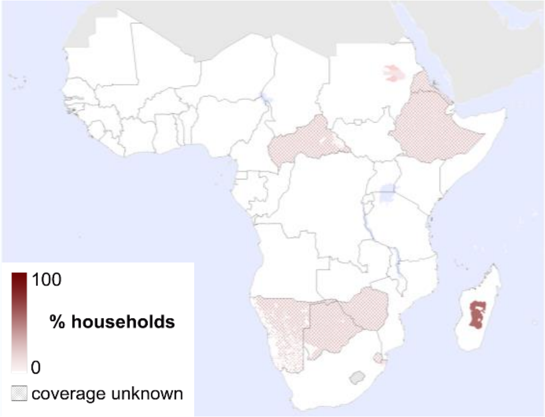

1999

carbamates

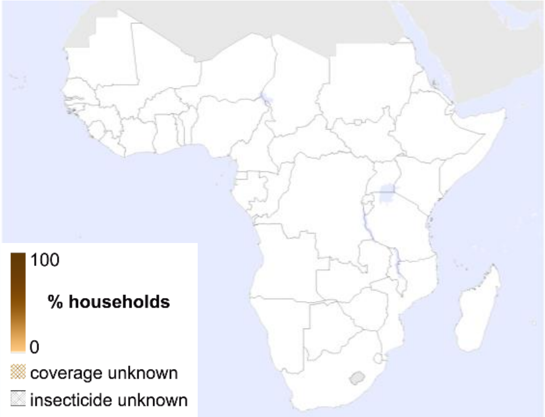

organochlorines

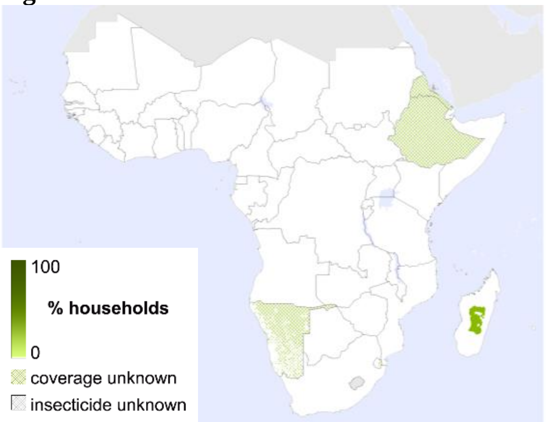

organophosphates

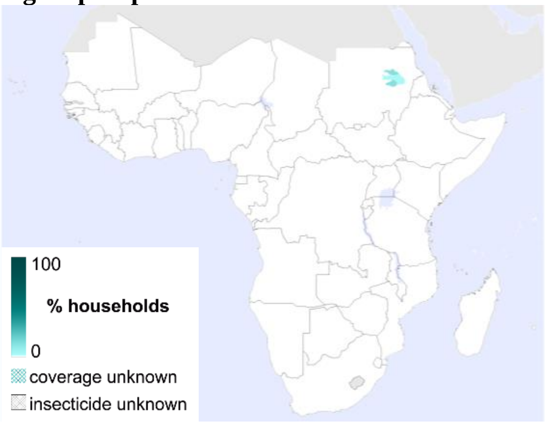

pyrethroids

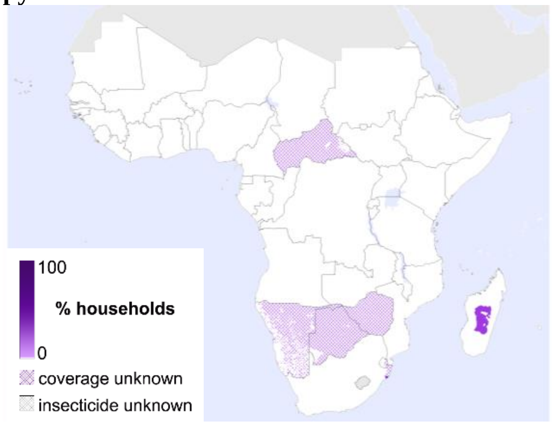

total

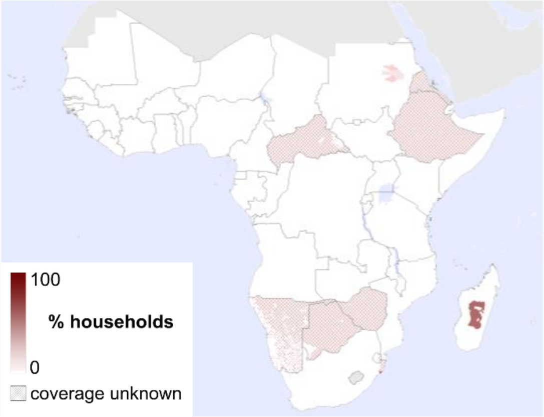

2000

carbamates

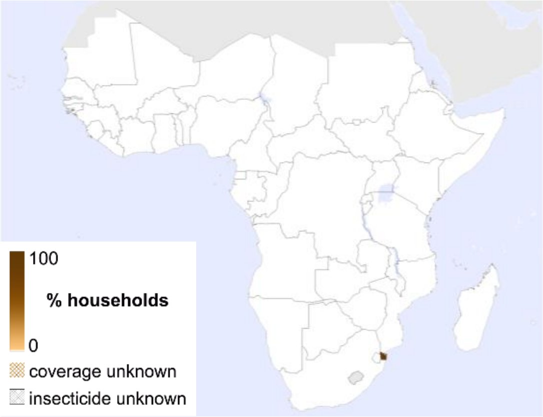

organochlorines

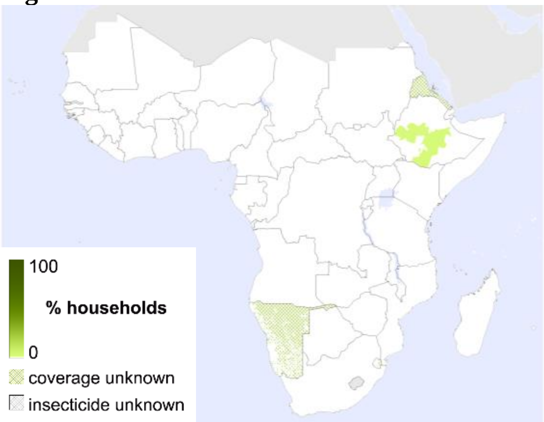

organophosphates

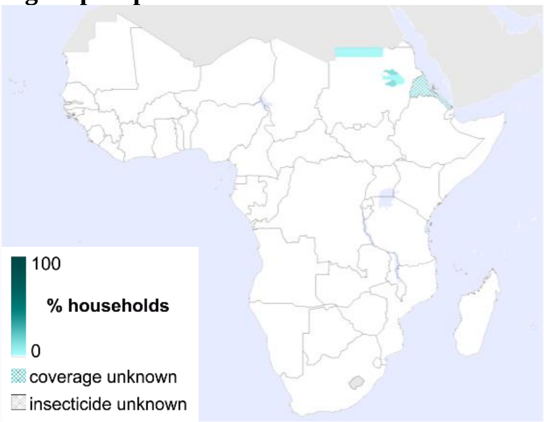

pyrethroids

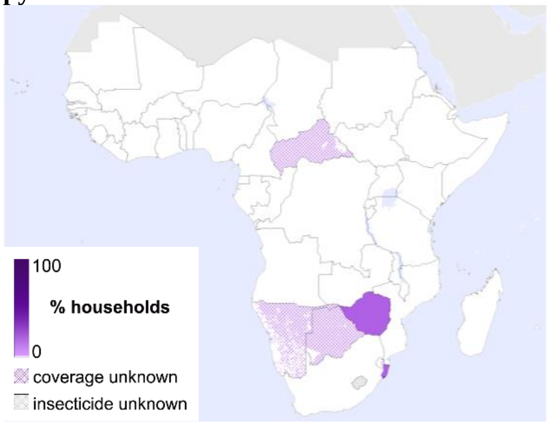

total

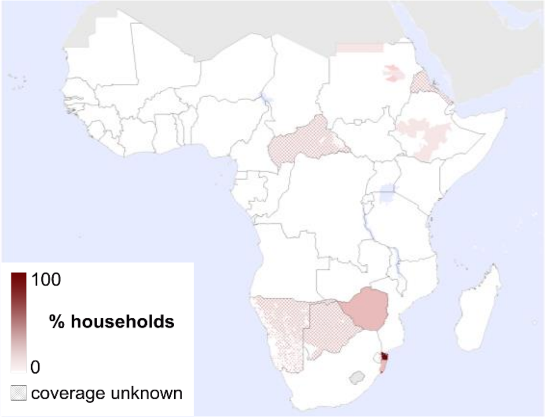

2001

carbamates

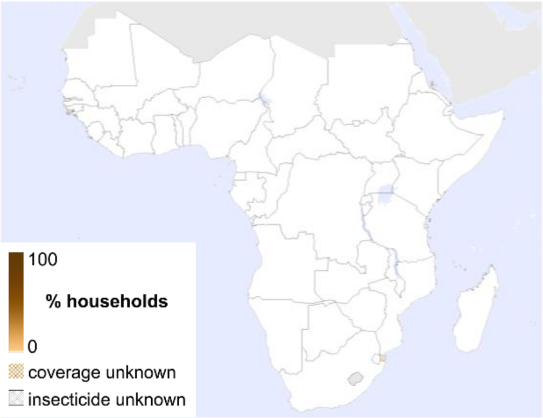

organochlorines

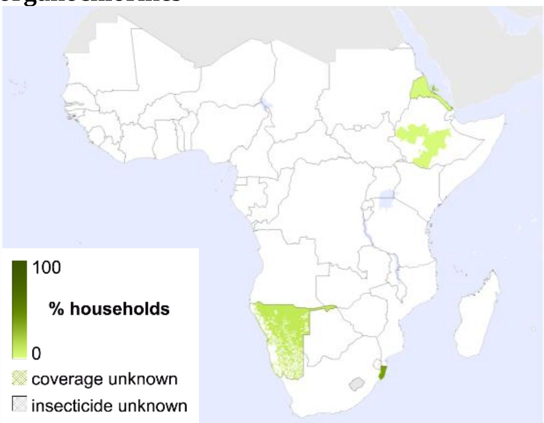

organophosphates

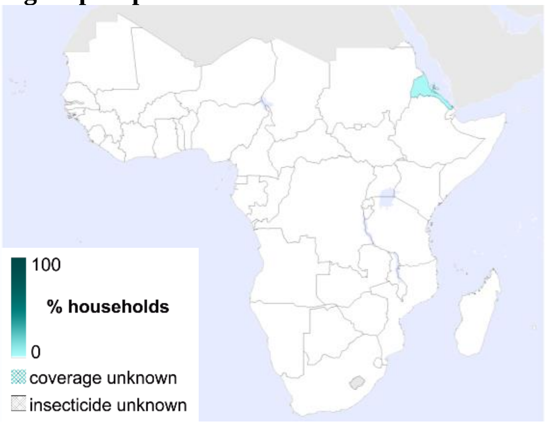

pyrethroids

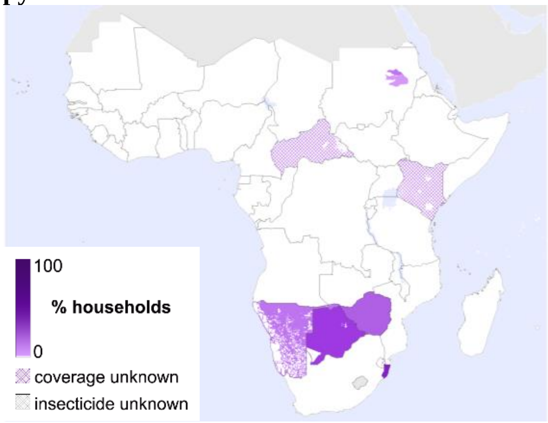

total

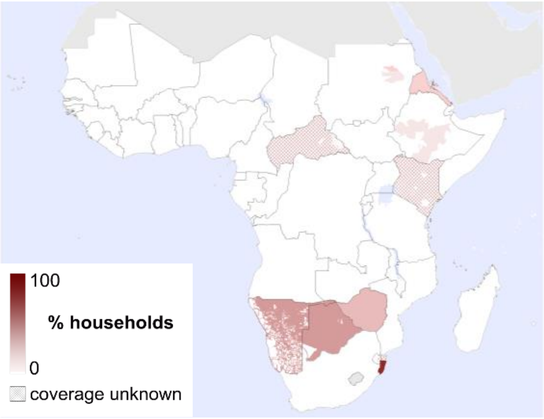

2002

carbamates

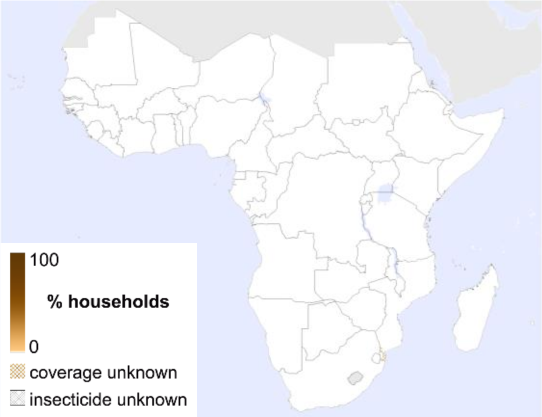

organochlorines

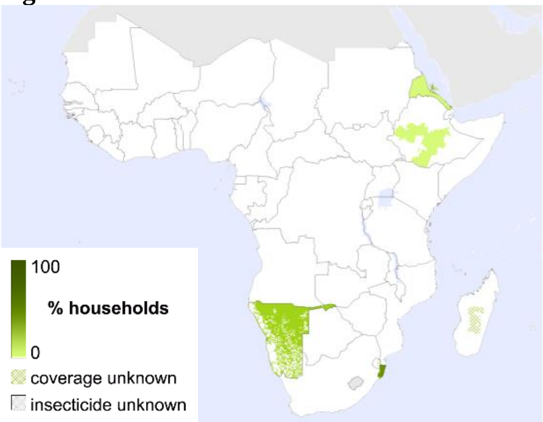

organophosphates

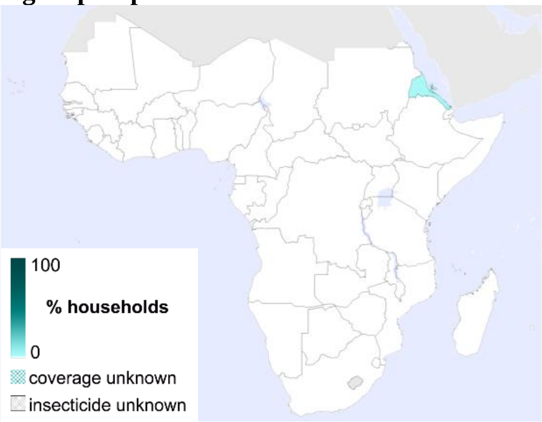

pyrethroids

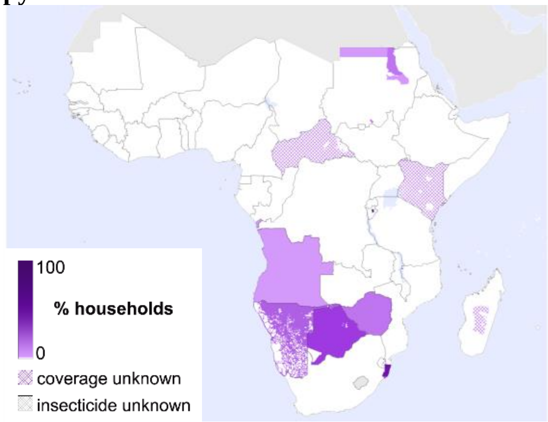

total

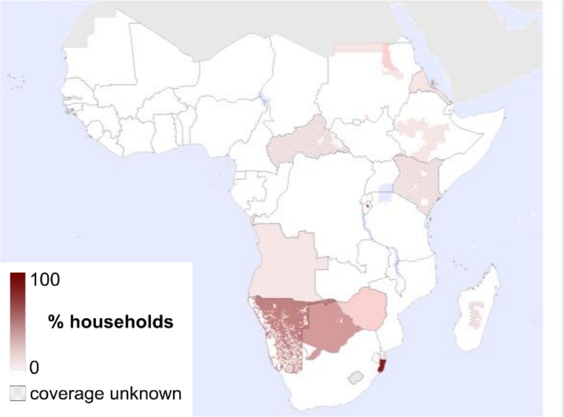

2003

carbamates

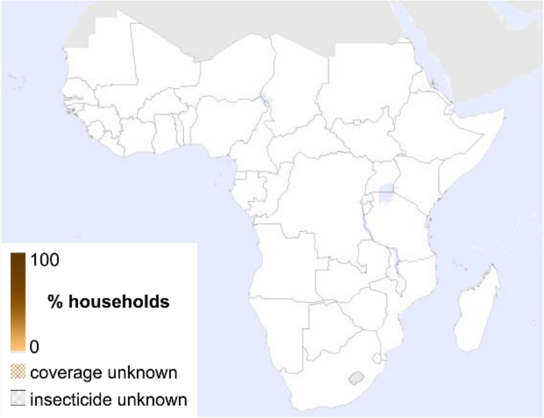

organochlorines

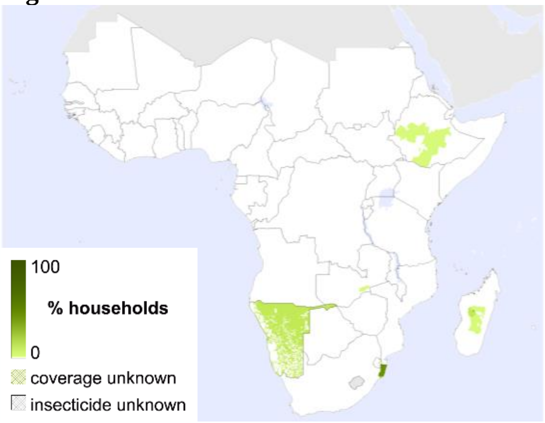

organophosphates

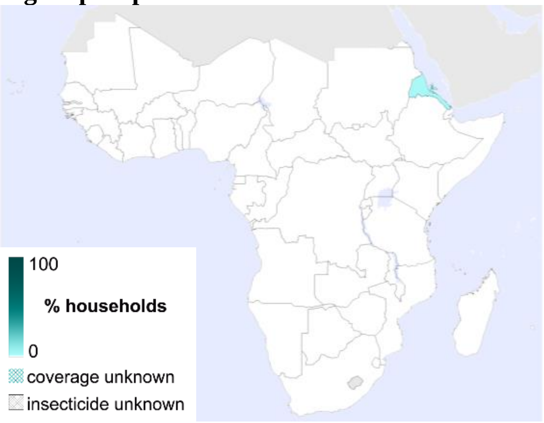

pyrethroids

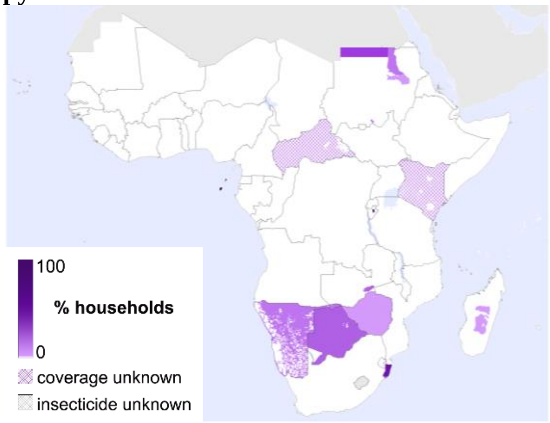

total

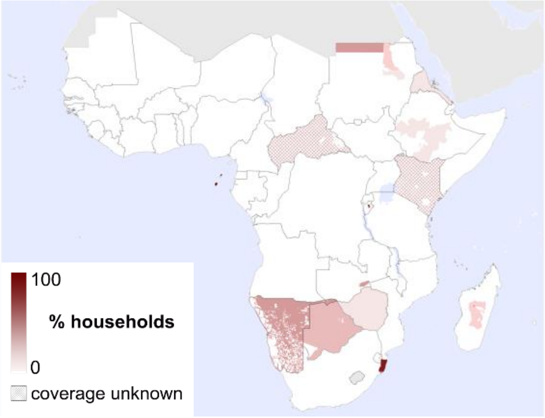

2004

carbamates

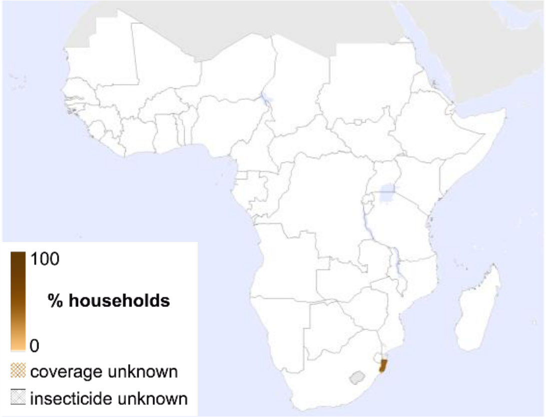

organochlorines

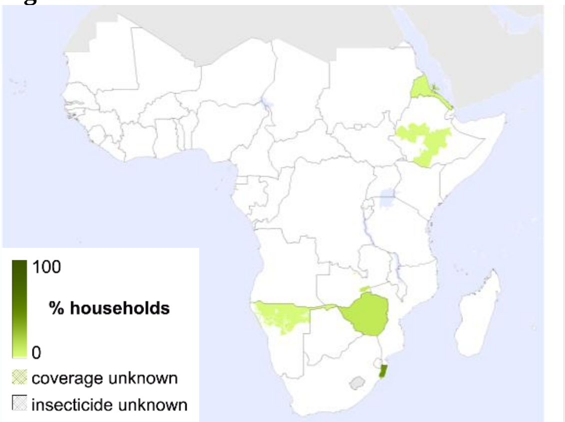

organophosphates

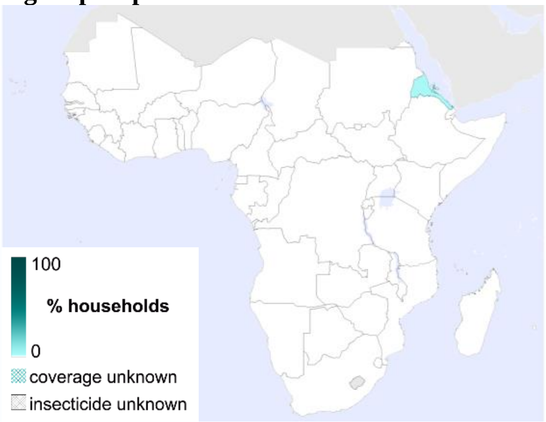

pyrethroids

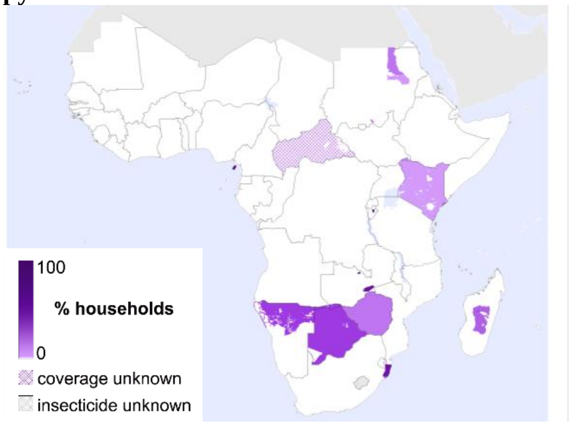

total

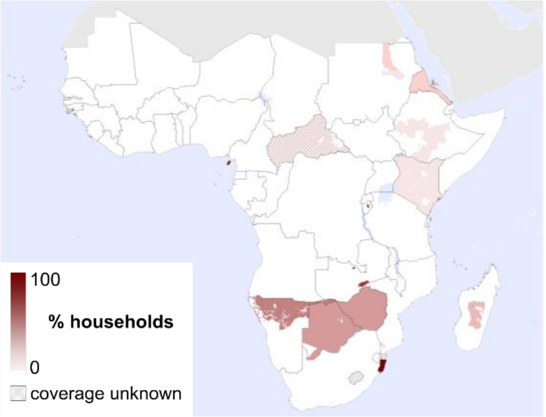

2005

carbamates

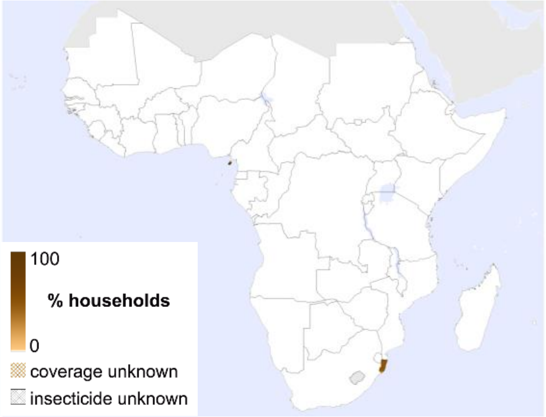

organochlorines

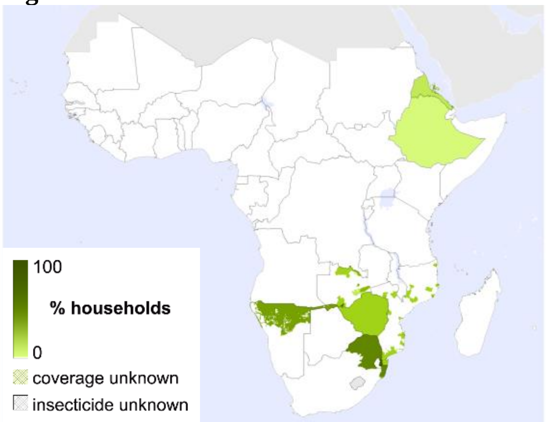

organophosphates

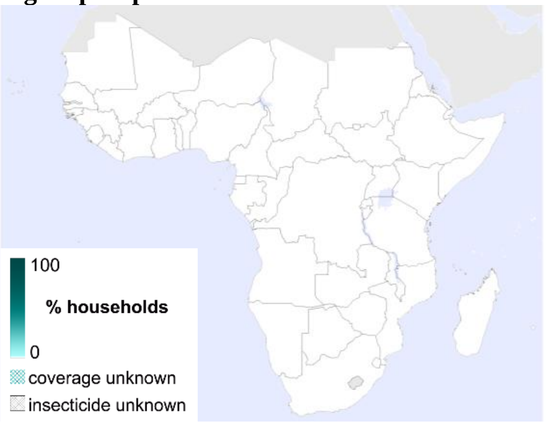

pyrethroids

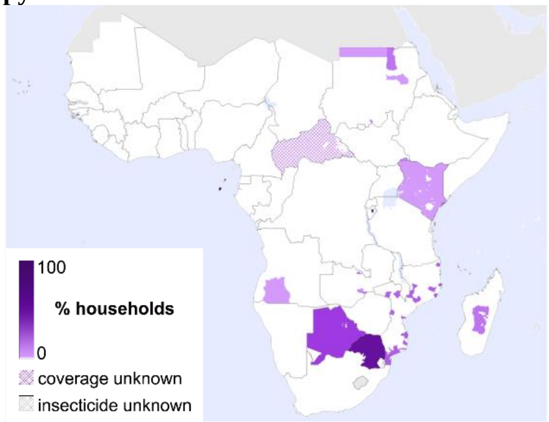

total

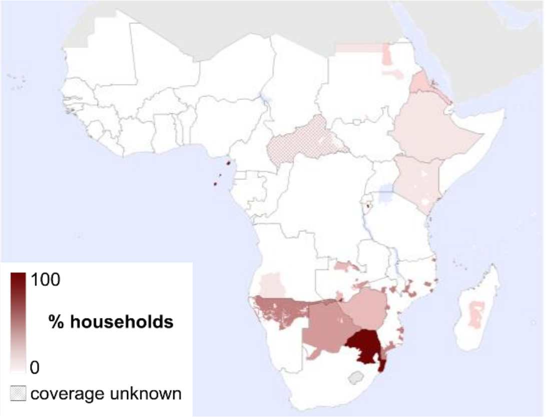

2006

carbamates

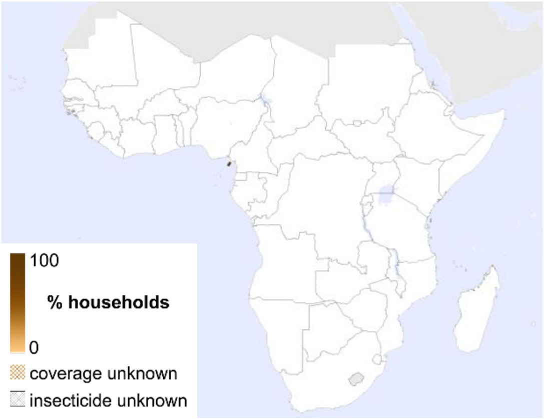

organochlorines

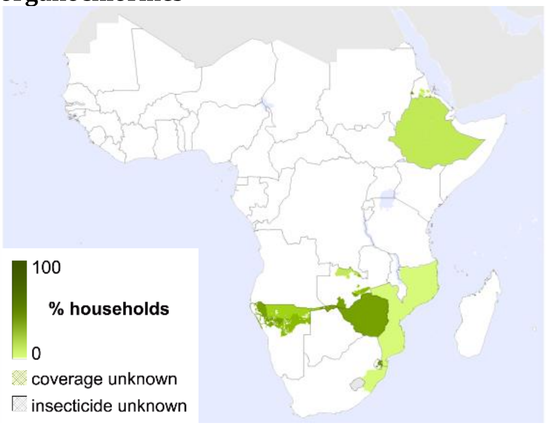

organophosphates

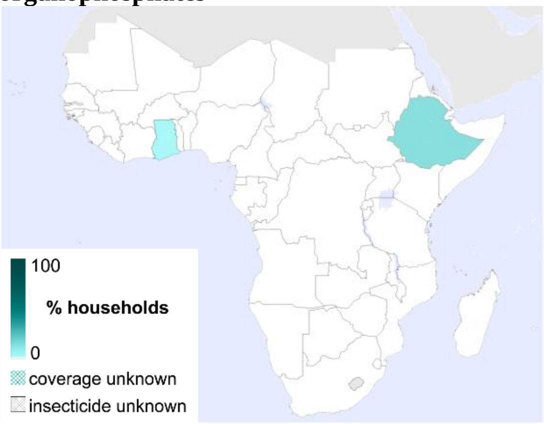

pyrethroids

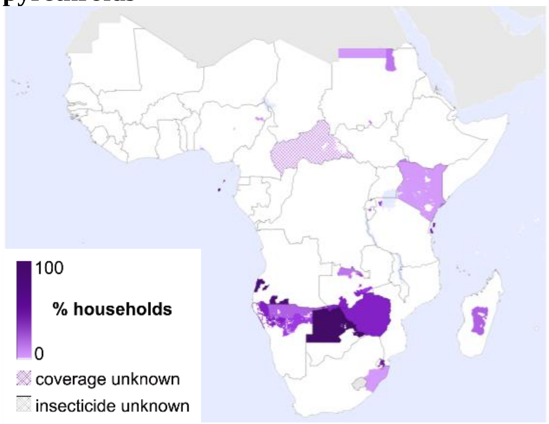

total

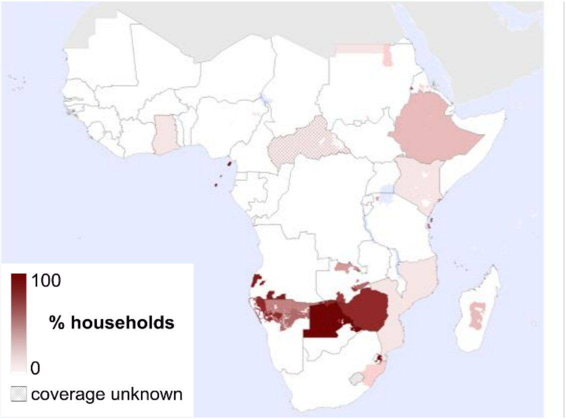

2007

carbamates

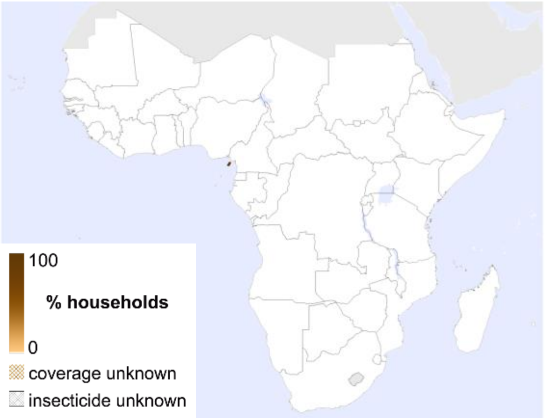

organochlorines

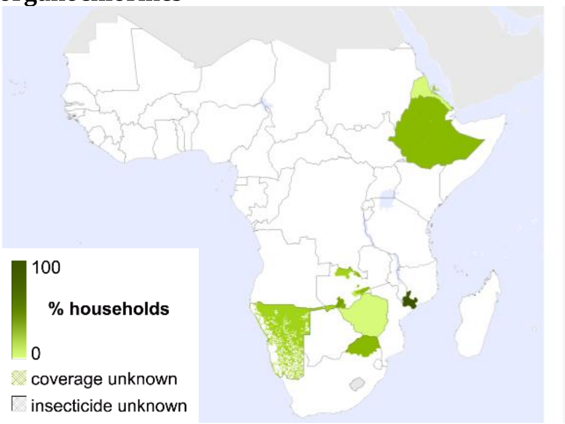

organophosphates

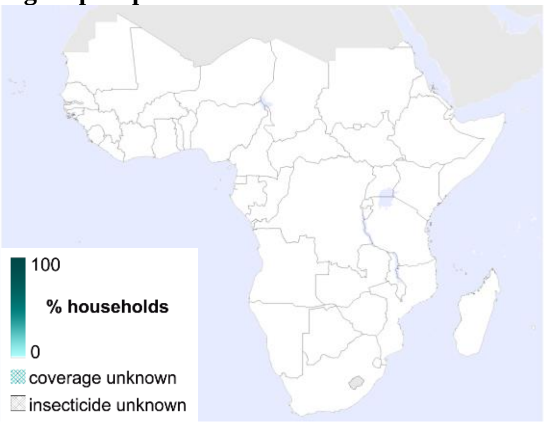

pyrethroids

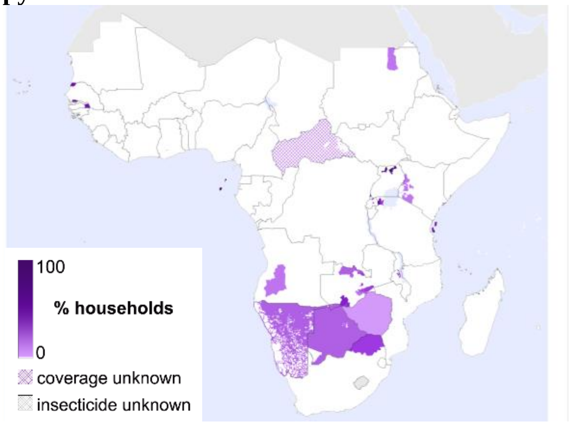

total

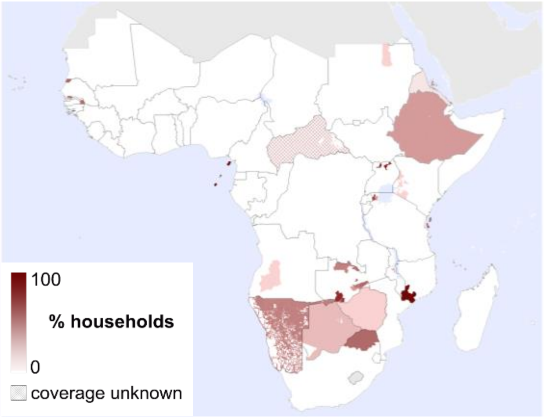

2008

carbamates

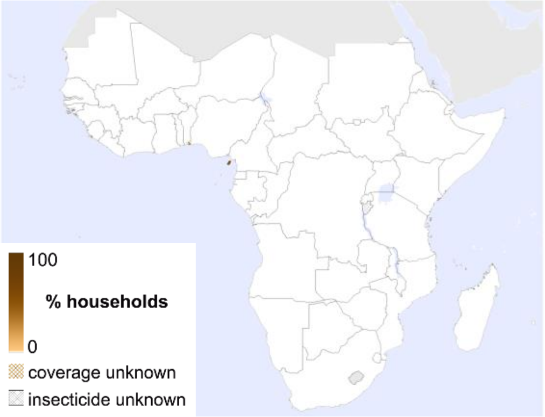

organochlorines

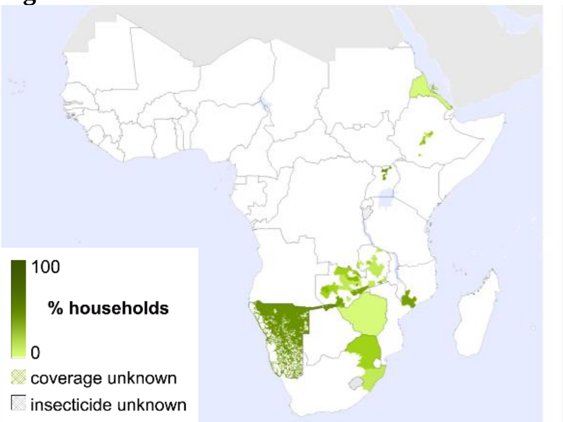

organophosphates

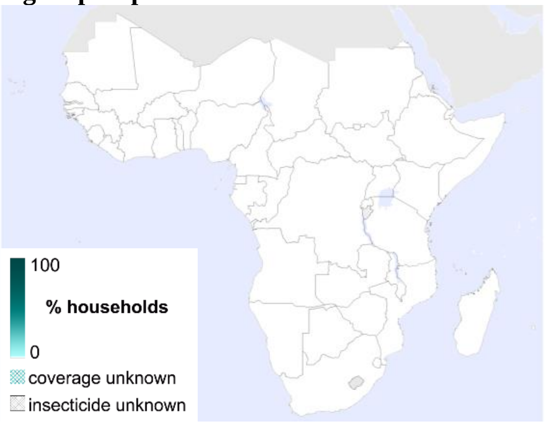

pyrethroids

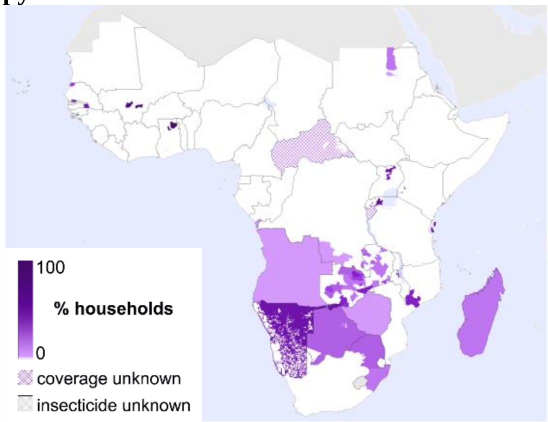

total

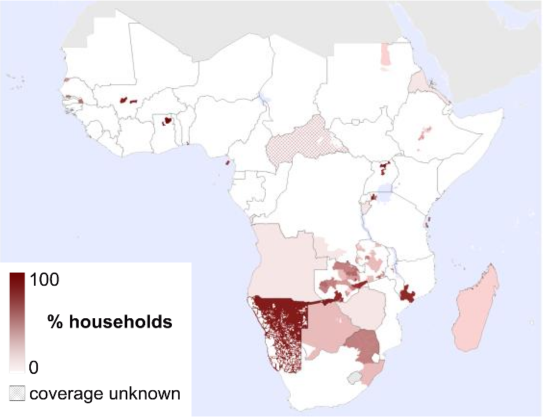

2009

carbamates

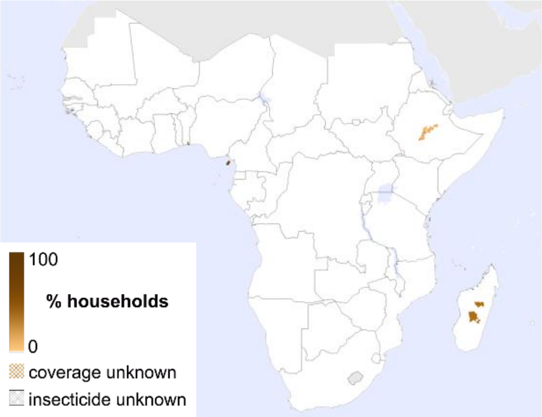

organochlorines

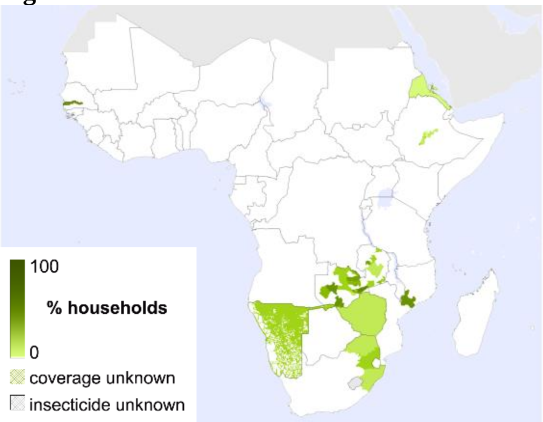

organophosphates

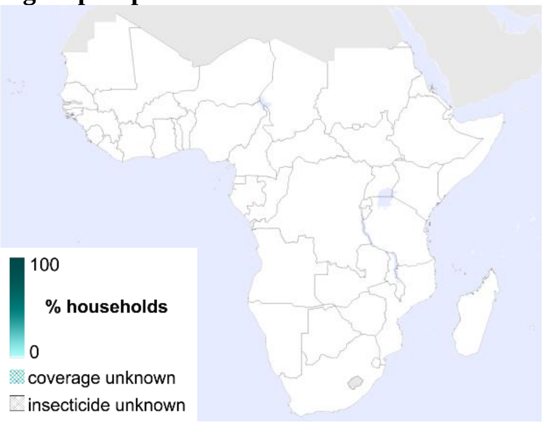

pyrethroids

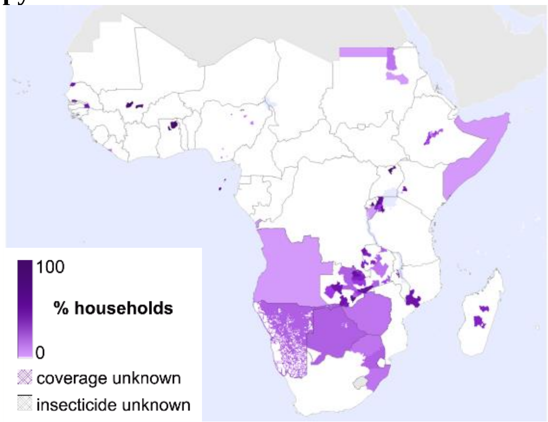

total

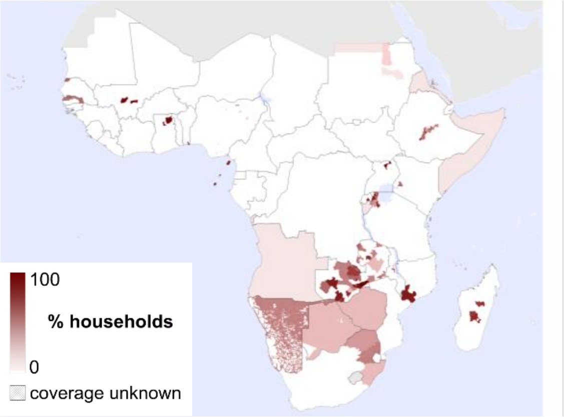

2010

carbamates

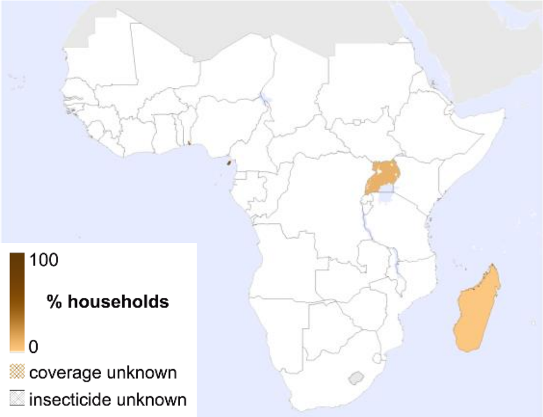

organochlorines

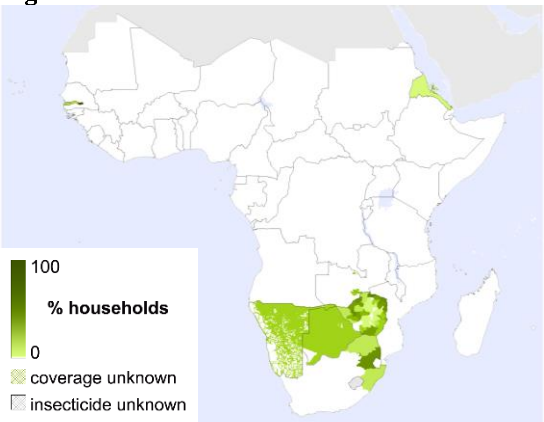

organophosphates

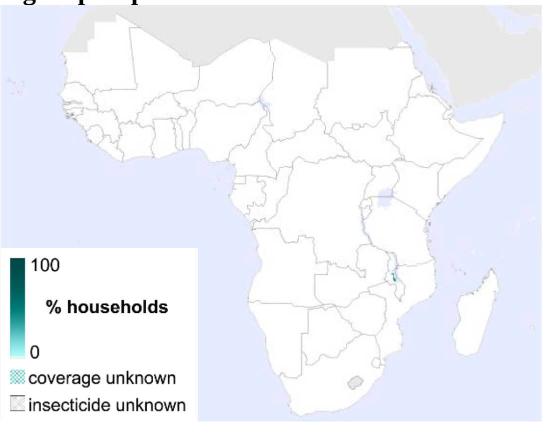

pyrethroids

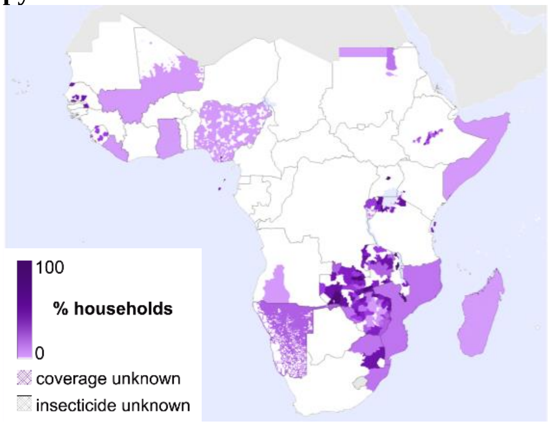

total

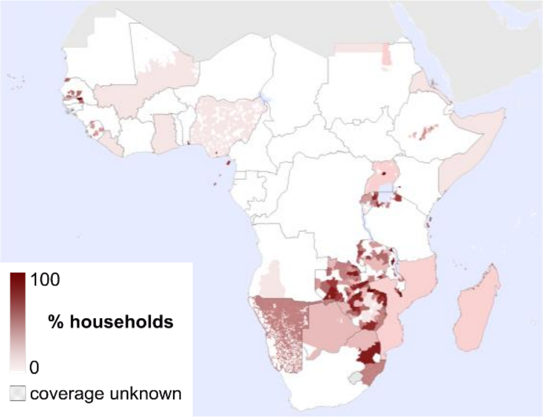

2011

carbamates

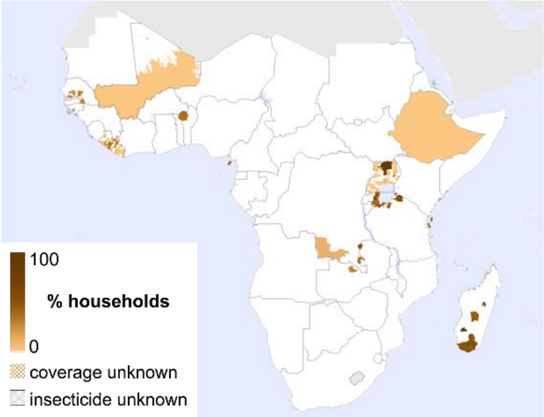

organochlorines

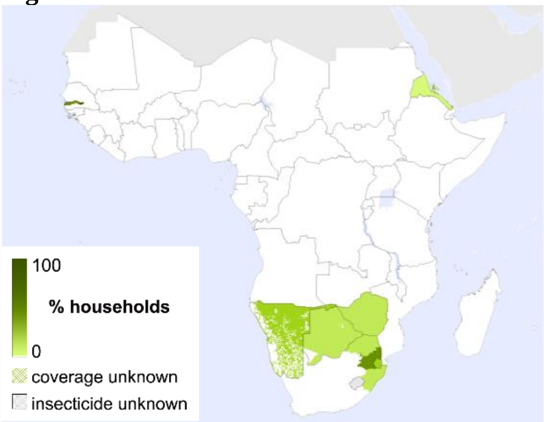

organophsophates

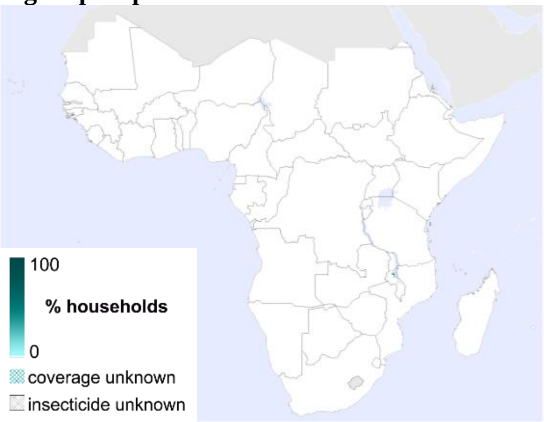

pyrethroids

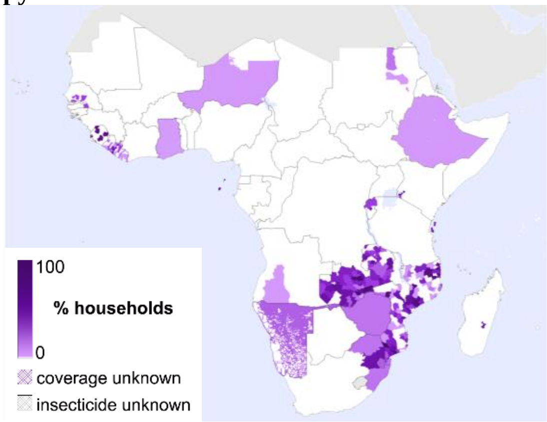

total

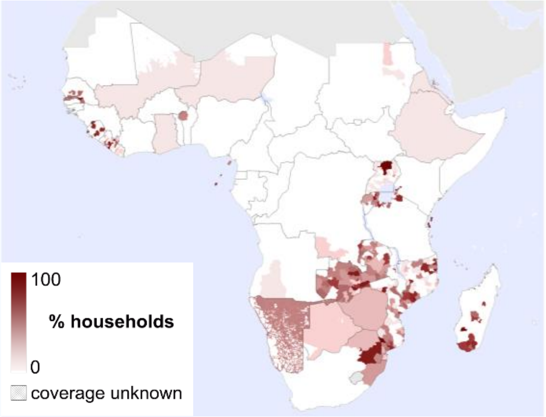

2012

carbamates

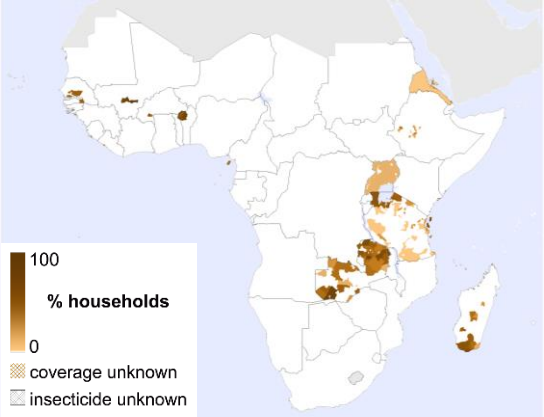

organochlorines

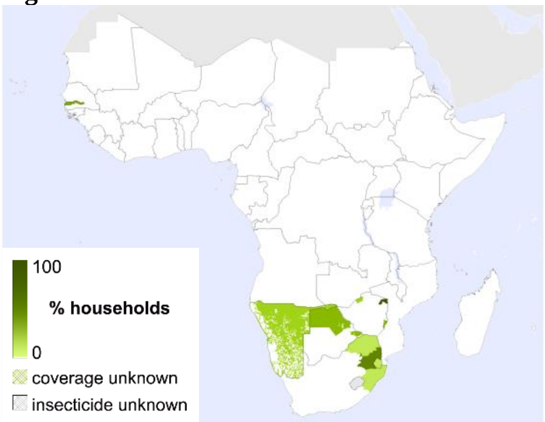

organophsophates

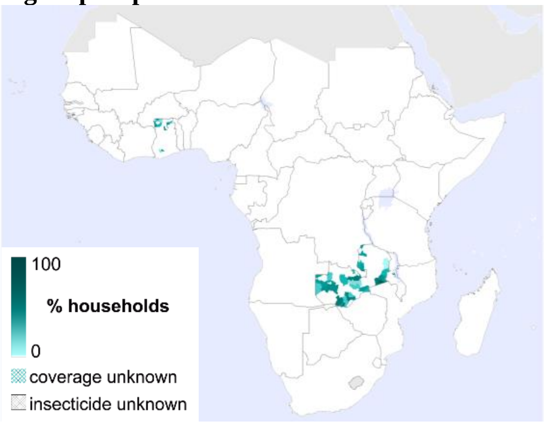

pyrethroids

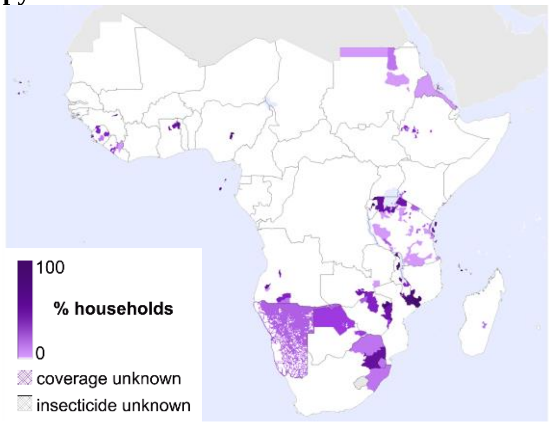

total

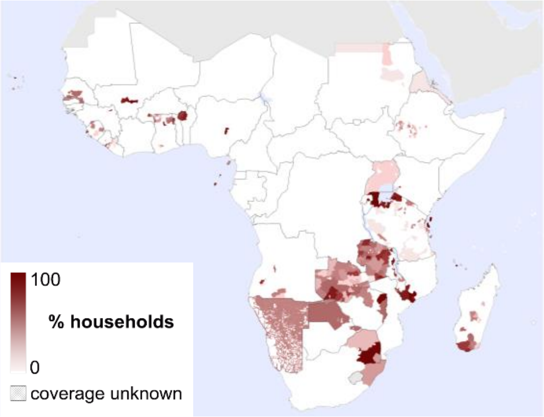

2013

carbamates

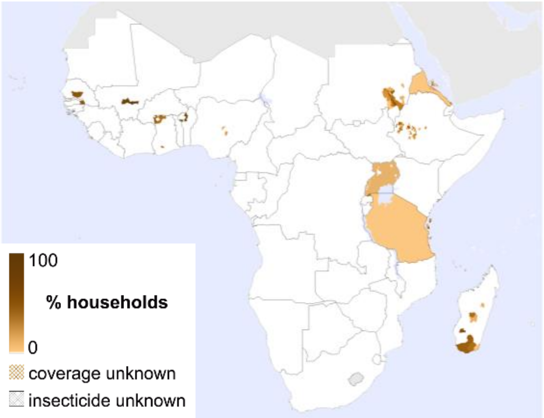

organochlorines

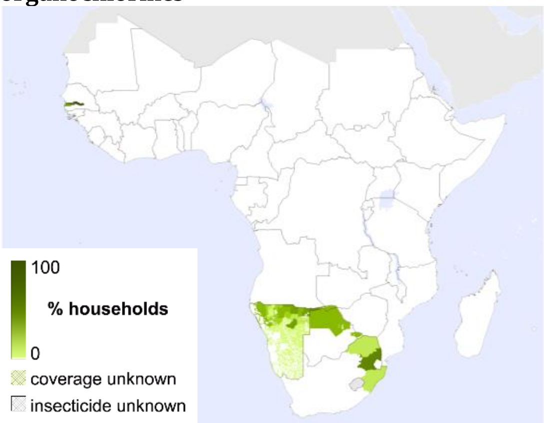

organophosphates

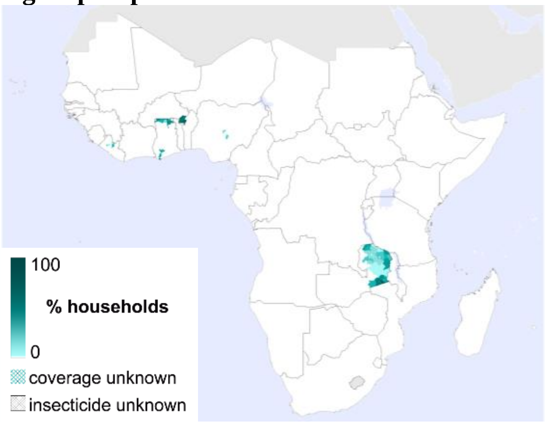

pyrethroids

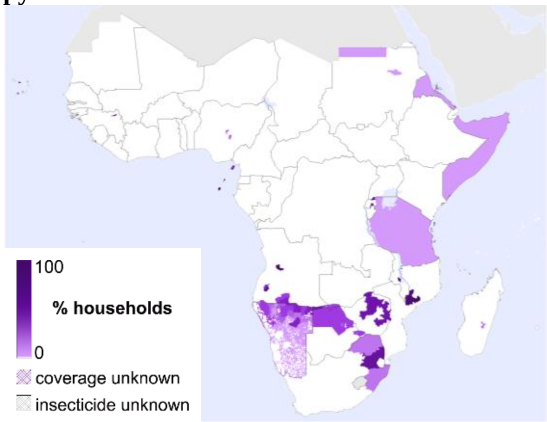

total

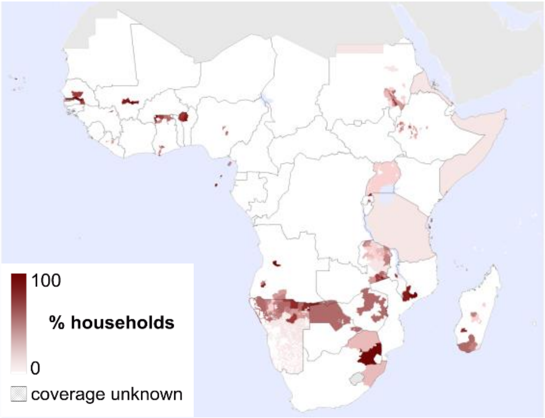

2014

carbamates

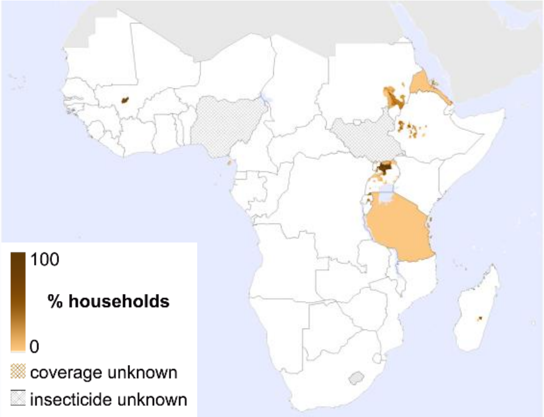

organochlorines

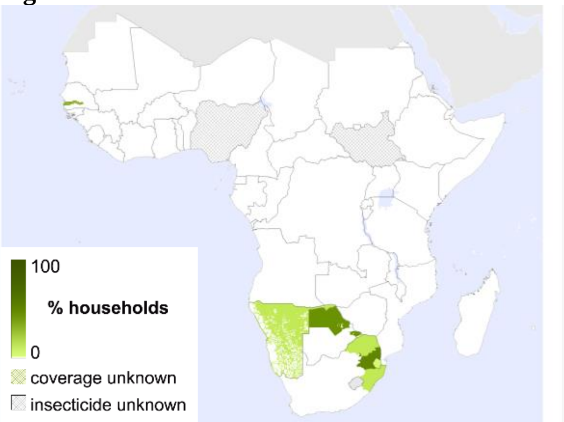

organophosphates

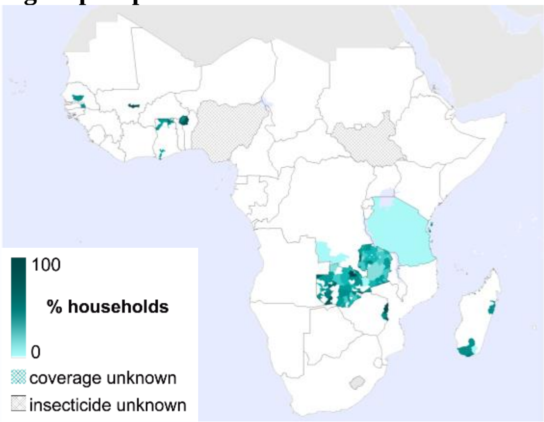

pyrethroids

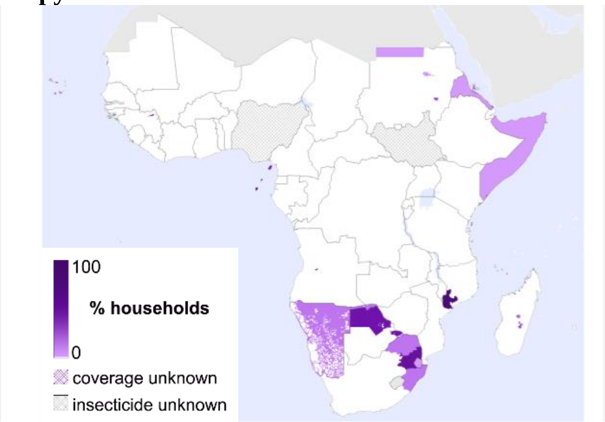

total

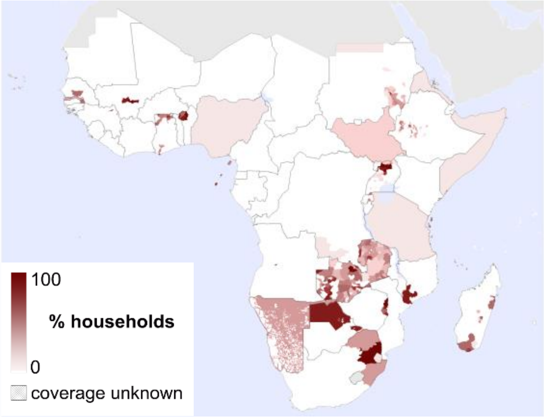

2015

carbamates

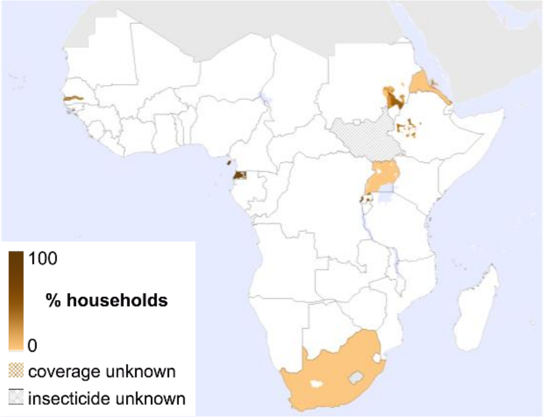

organochlorines

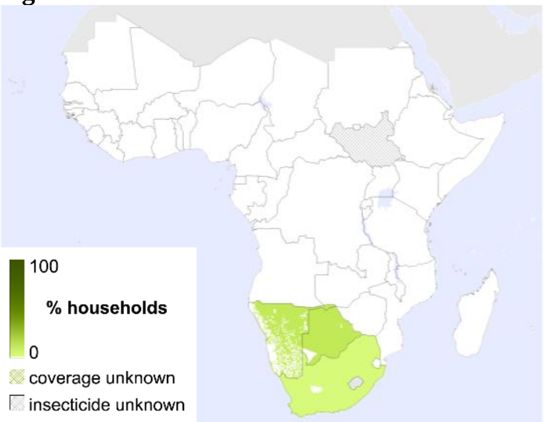

organophosphates

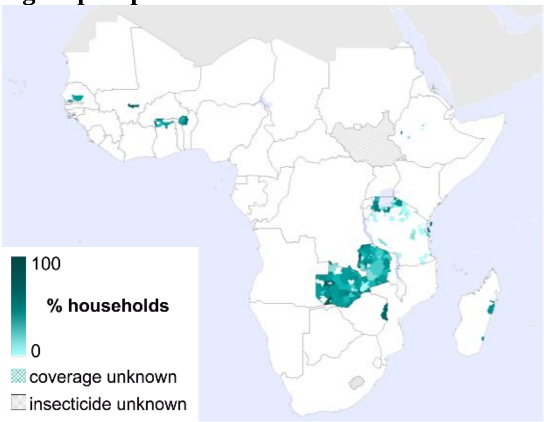

pyrethroids

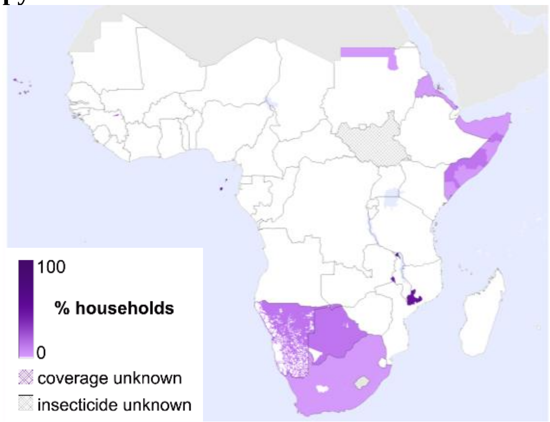

total

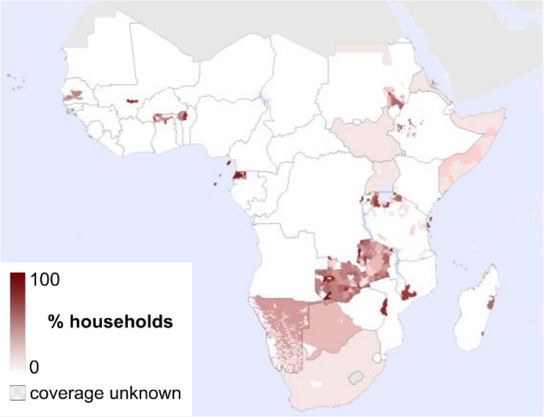

2016

carbamates

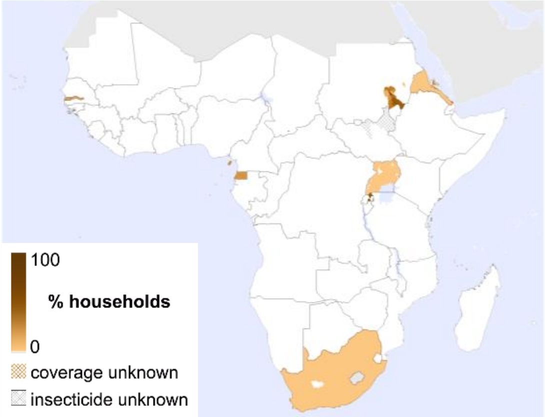

organochlorines

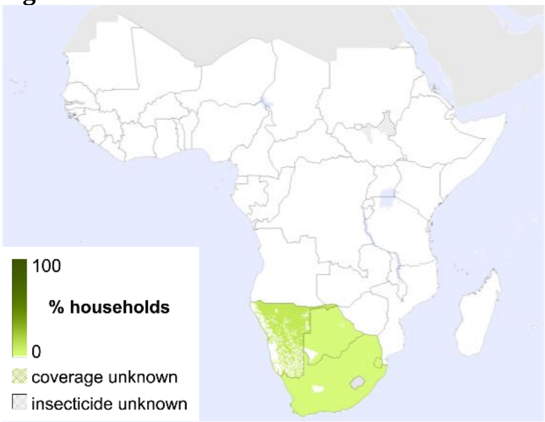

organophosphates

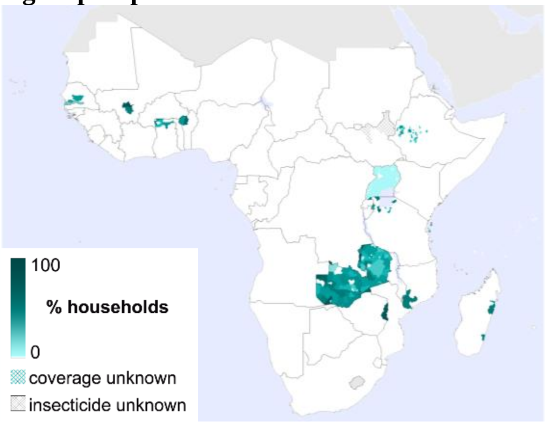

pyrethroids

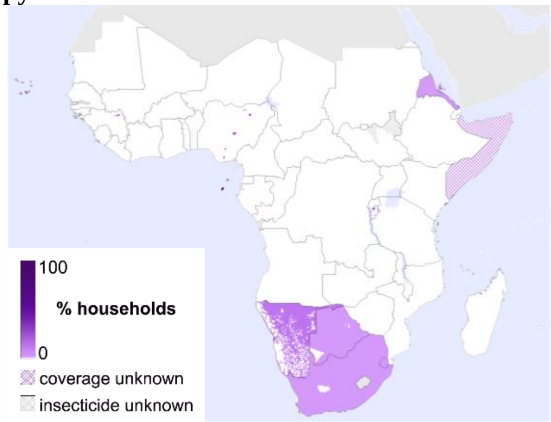

total

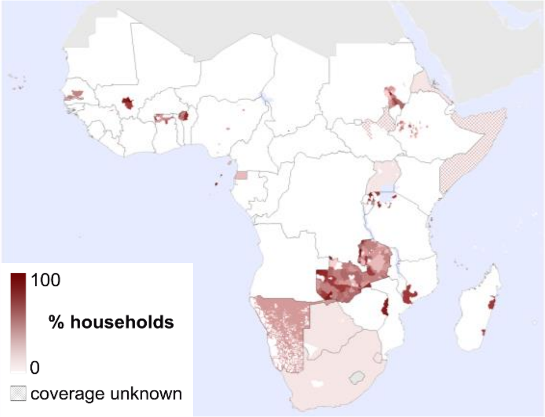

2017

carbamates

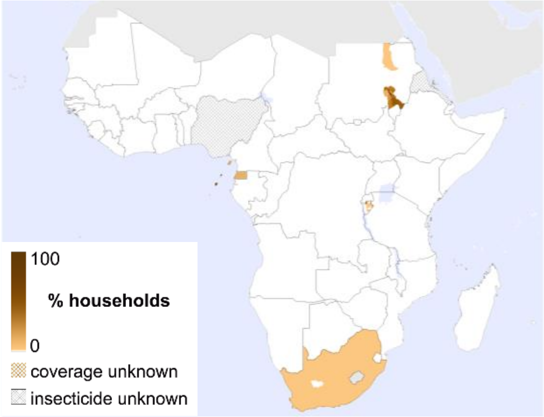

organochlorines

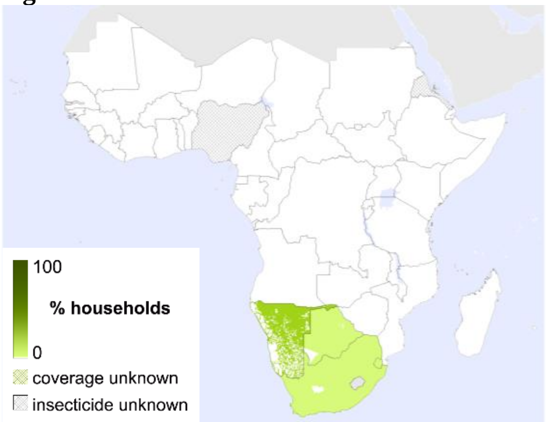

organophosphates

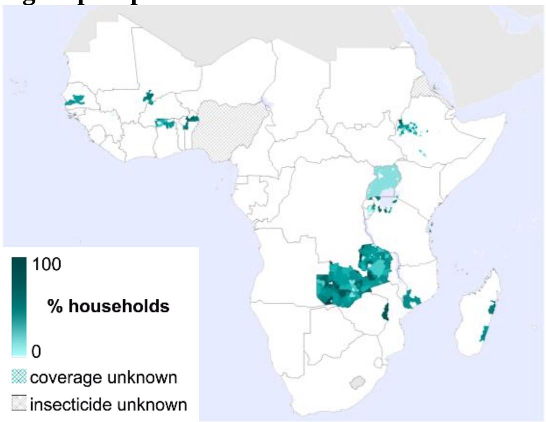

pyrethroids

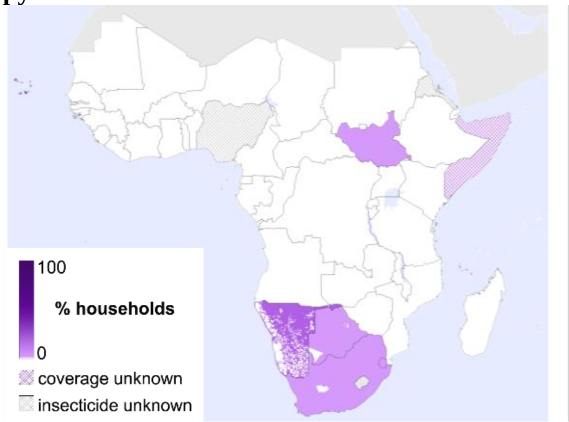

total

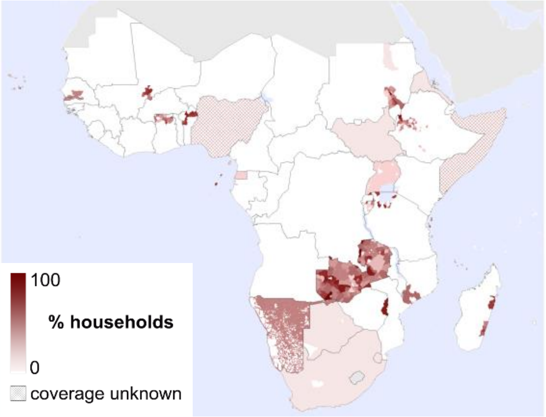

Supplement: Supplementary file 1 — Additional file 1: Maps showing IRS coverage in sub-Saharan Africa from 19972017 for carbamates, organochlorines, organophosphates and pyrethroids. [file 12936_2020_3216_MOESM1_ESM.pdf]
