# Supplementary figures and images for: Indoor residual spraying for malaria control in sub-Saharan Africa 1997 to 2017: an adjusted retrospective analysis
Source: Malar J. 2020 Apr 10;19:150. doi: 10.1186/s12936-020-03216-6 (PMC7149868; doi:10.1186/s12936-020-03216-6)

**Maps showing modelled insecticide-treated bed net (ITN) coverage from Bhatt and Gething, 2014**

**2000**

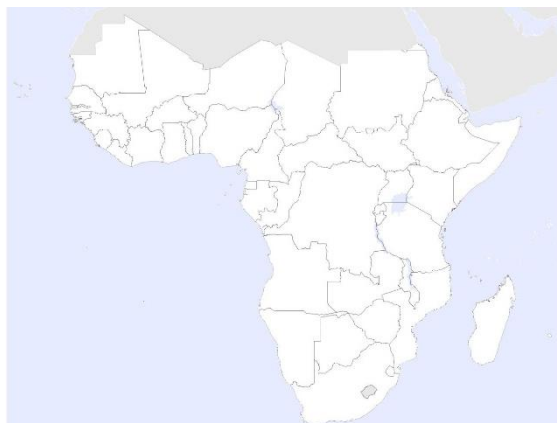

**2005**

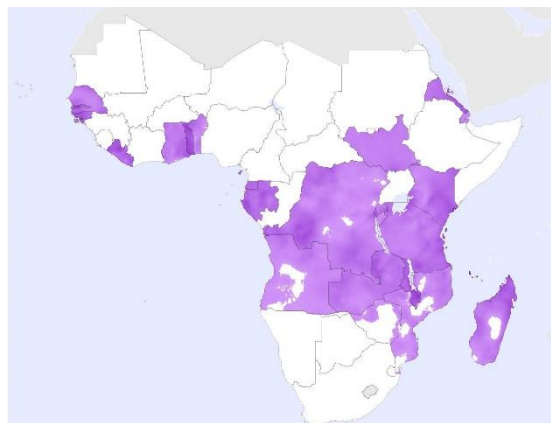

**2007**

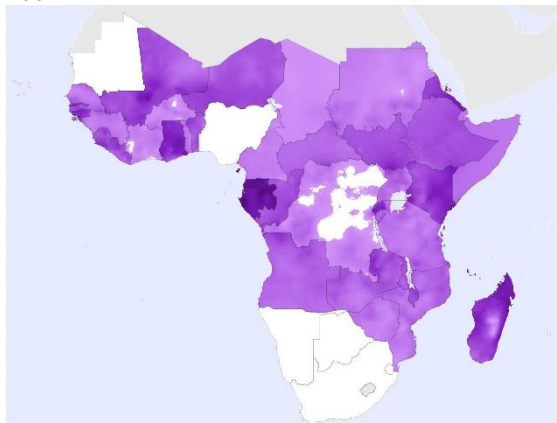

**2010**

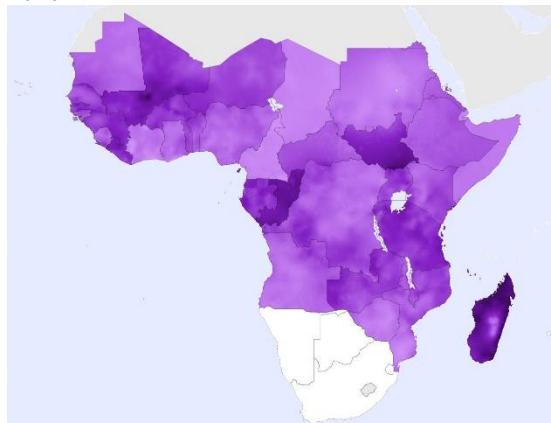

**2015**

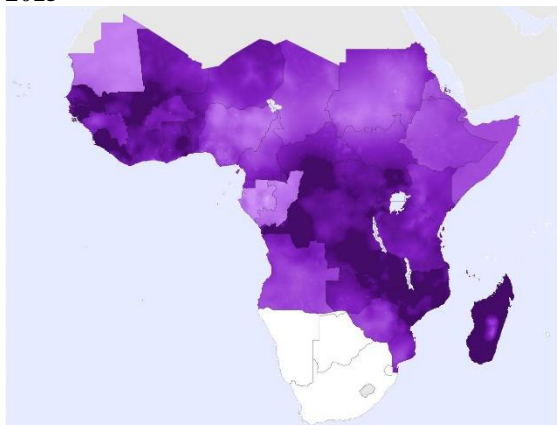

Supplement: Supplementary file 2 — Additional file 2: Maps showing modelled insecticide-treated bed net (ITN) coverage from Bhatt and Gething [35]. [file 12936_2020_3216_MOESM2_ESM.pdf]

***Plasmodium falciparum* infection prevalence from Bhatt *et al.*, 2015**

**2000**

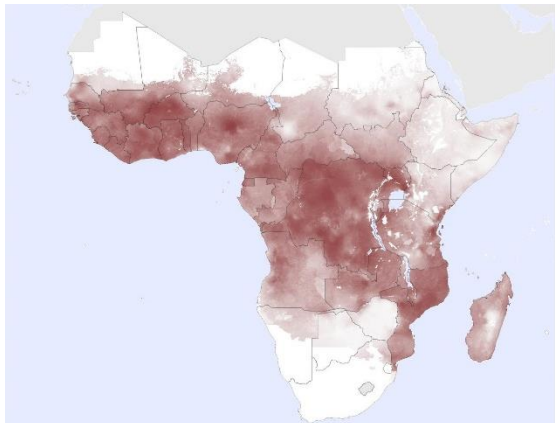

**2005**

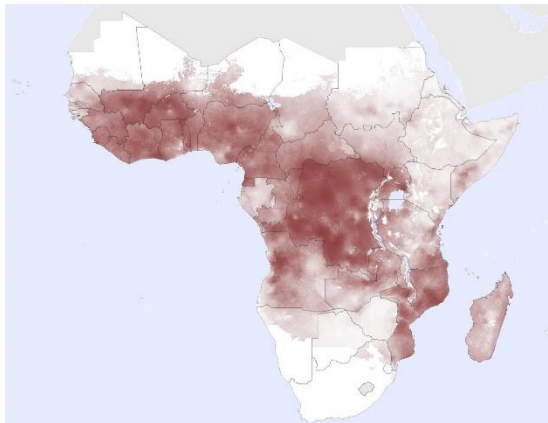

**2007**

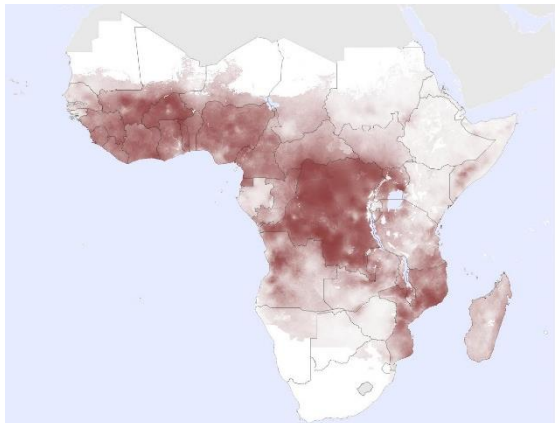

**2010**

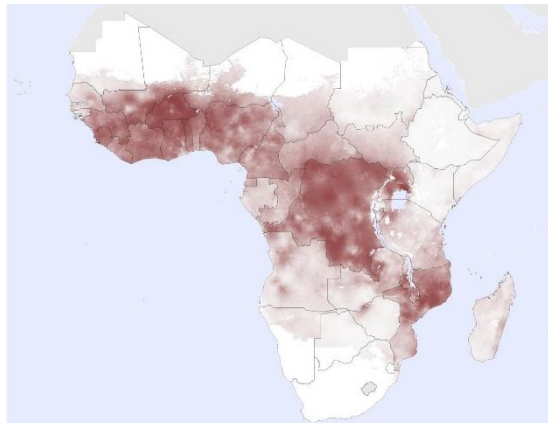

**2015**

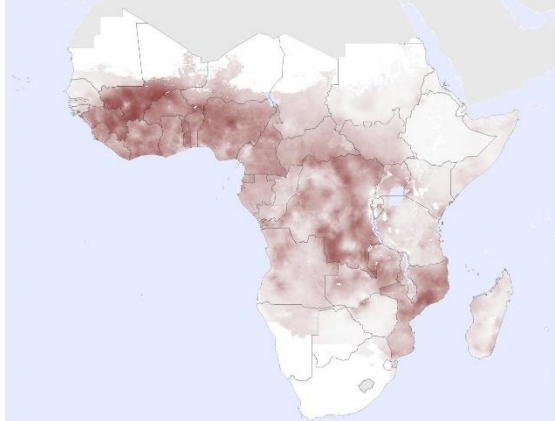

Supplement: Supplementary file 3 — Additional file 3:Plasmodium falciparum infection prevalence from Bhatt et al. [1]. [file 12936_2020_3216_MOESM3_ESM.pdf]
